# Delineating transcriptomic signatures of *in vitro* human skeletal muscle models in comparison to *in vivo* references

**DOI:** 10.1016/j.stemcr.2025.102684

**Published:** 2025-10-23

**Authors:** Margaux Van Puyvelde, Eslam Essam Mohammed, Ángela Moreno Anguita, Jarne Bonroy, Sandra Jansen, Atilgan Yilmaz

**Affiliations:** 1 Leuven Stem Cell Institute, Department of Development and Regeneration, KU Leuven, 3000 Leuven, Belgium

**Keywords:** skeletal muscle, *in vitro* models, skeletal muscle differentiation, skeletal muscle transdifferentiation, transcriptomics, bulk RNA sequencing, single cell RNA sequencing

## Abstract

A pivotal question at the heart of stem cell research is how faithful cellular models recapitulate human tissues. Skeletal muscle, the largest organ in the human body, has been modeled by various *in vitro* systems. Here, we sought to delineate the state-of-the-art of muscle models by performing a large-scale analysis of transcriptome datasets, covering over 400 samples across 39 studies, including bulk and single-cell RNA sequencing of 2D and 3D models and their *in vivo* counterparts. By comparing these models to *in vivo* muscle, we highlighted failed upregulation of myogenic factors and retention of epigenetic memory from the *in vitro* source material. We featured differences in lipid metabolism and depletion of multiple fibroblast growth factor (FGF) ligands in the *in vitro* models. Finally, we revealed model-dependent variation in myogenic progenitors. Our analyses highlight targetable processes to improve the models while paving the way for similar studies on other cell types.

## Highlights

• *In vitro* skeletal muscle models retain epigenetic memory from their source material
• hPSC-derived and transdifferentiation models differ from *in vivo* in lipid metabolism
• Transdifferentiation model fails to induce specific myogenic regulatory factors
• 2D and 3D models generate PAX7^+^ cells at different degrees of quiescence

## Graphical abstract

**Figure:**
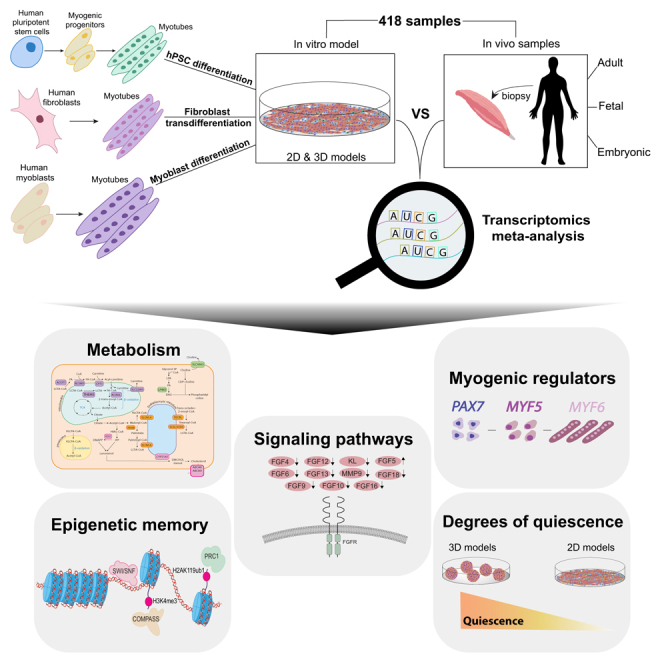

## Teaser

In this article, Yilmaz and colleagues perform a large-scale transcriptomics metaanalysis with over 400 samples comparing established *in vitro* skeletal muscle models to *in vivo* skeletal muscle samples from adult, embryonic, and fetal biopsies. They highlight differences across bulk and single-cell RNA sequencing datasets in expression of transcription factors, epigenetic complexes, metabolic processes, signaling pathways and in quiescence signatures.

## Introduction

The use of reliable *in vitro* models is essential to study human development, disease, regeneration, and therapeutic interventions. Methods of model generation vary from the differentiation of human pluripotent stem cells (hPSCs) to transdifferentiation of somatic cells or the use of primary isolated and immortalized cell types (Chal et al., 2016; Kim et al., 2018; Weintraub et al., 1989). Despite the great technical advantages and a vast number of insights they have given, all these cellular models have their own challenges. Primary cells quickly lose many of their *in vivo* characteristics and after a prolonged period of culturing, will enter senescence, while immortalization alters their expression patterns (Deng et al., 2020). Alternatively, differentiation protocols from hPSCs are often long and expensive and transdifferentiation from somatic cells typically exhibits low conversion efficiency with partial retention of the epigenetic profile of the source cells (Manandhar et al., 2017; Shahriyari et al., 2022). Moreover, cells obtained through differentiation and transdifferentiation methods have been described to be more immature (Ottaviani et al., 2023).

Skeletal muscle is the largest tissue, encompassing about 40 percent of the human body mass (Frontera and Ochala, 2015). While providing the mechanism behind movement, it also plays an essential role in metabolism and immune functions. Skeletal muscle is susceptible to a plethora of genetic and metabolic disorders and undergoes wasting in cancer and upon aging, making this tissue a prime target of regenerative medicine. Having 2D and 3D models that can faithfully recapitulate human muscle is crucial to get insight into its development, diseases, and regeneration and to aid the identification of novel therapies.

A growing body of research has been dedicated to the analysis of skeletal muscle models and biopsies through the lens of transcriptomics. While most of the efforts were focused on bulk RNA sequencing (RNA-seq), a smaller and more recent pool of studies made use of single-cell or single-nucleus RNA (scRNA and snRNA) sequencing. Transcriptome analysis has been instrumental in elucidating developmental trajectories and disparities between healthy and diseased muscle tissue. Nevertheless, to date, skeletal muscle transcriptomic data have not been utilized to discern molecular differences between the *in vitro* models and bona fide skeletal muscle samples in a systematic way.

In this study, we bring together 39 bulk RNA-seq and scRNA-seq studies covering over 400 samples from all types of *in vitro* skeletal muscle models and compare these to different stages of human adult and fetal muscle biopsies (Banerji et al., 2020; Bargiela et al., 2019; Batra et al., 2017; Benarroch et al., 2023; Bernstein et al., 2010; Bisceglie et al., 2021; Cerro-Herreros et al., 2021; Choi et al., 2020; 2016; Dall’Agnese et al., 2019; Dunham et al., 2012; Franco et al., 2019; Hicks et al., 2018; Jaime et al., 2023; Kabadi et al., 2015; Kayman Kürekçi et al., 2022; Lim et al., 2021; Lucas et al., 2018; Manandhar et al., 2017; Marg et al., 2019; Mavrommatis et al., 2023; Nayak et al., 2021; Polstein et al., 2017; Raue et al., 2024; Resnick et al., 2019; Rossi et al., 2023; Rubenstein et al., 2020; Shadle et al., 2019; Somers et al., 2022; Stearns-Reider et al., 2023; Di Stefano et al., 2019; Todorow et al., 2021; van der Wal et al., 2018; Wang et al., 2022; Wood et al., 2021; Wu et al., 2018; Xi et al., 2020) (Data S1: GEO accession). We show failure of expression in several myogenic factors, aberrant transcription factor signatures, epigenetic memory retention, and major differences in fatty acid metabolism and membrane transporter expression patterns in different *in vitro* models. Additionally, upon integration of 6 scRNA-seq datasets, we highlight a continuum of quiescence across different 2D and 3D methods of generation of myogenic progenitors and reveal a potential role for the BRCA1-BRCA2-containing complex in proliferating developmental human myogenic progenitors. Our analyses shed light on the common discrepancies between *in vitro* models and bona fide skeletal muscle cells across different cellular processes and provide a reference for future studies to improve the existing models.

## Results

### Large-scale analysis of bulk RNA sequencing samples reveals differences between *in vitro* models and bona fide skeletal muscle

**Figure 1:**
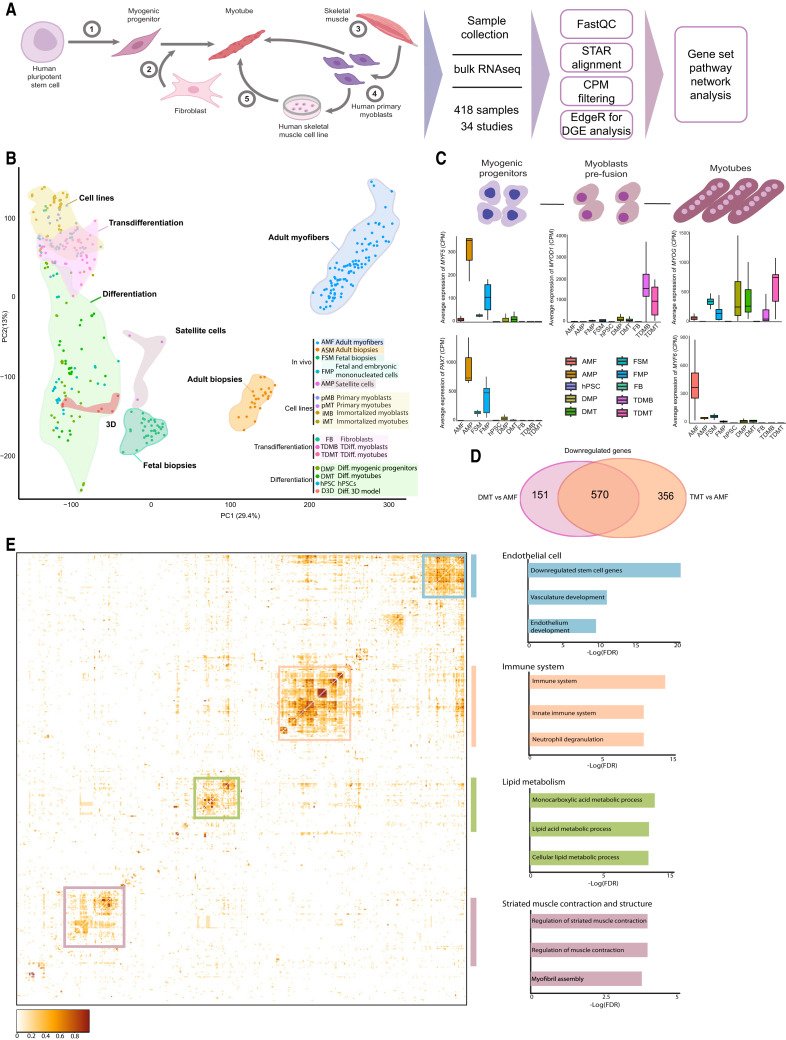
Transcriptome-wide comparison and myogenic profiles of the integrated *in vitro* and *in vivo* skeletal muscle samples (A) Schematic overview of sample collection and analysis pipeline. (B) Principal-component analysis (PCA) for all collected samples included in the study. (C) Average counts per million (CPM) values of the myogenic regulatory factors (MRFs) for their respective stages depicted in the illustration for human pluripotent stem cell (hPSC)-derived differentiated myogenic progenitors (DMPs) and myotubes (DMTs), transdifferentiated myoblasts (TDMBs) and myotubes (TDMTs), fibroblasts (FBs), hPSCs, adult myogenic progenitors (AMPs), and adult isolated myofibers (AMFs), and fetal skeletal muscle biopsies (FSMs) and fetal myogenic progenitors (FMPs). Data are presented as the mean ± SE. (D) Venn diagram showing the overlap in significantly downregulated genes (FDR< 0.05) between DMT and TDMT as compared to AMF. (E) Heatmap demonstrating predicted protein-protein interaction network scores for the commonly downregulated genes in (D). Predicted interaction values represent a confidentiality score between 0 and 1, as depicted in the scale bar. Clusters are analyzed through gene set enrichment analysis (GSEA) and are summarized in the bar plots on the right of the heatmap. For each gene ontology term with significance cut-off of *p* < 0.05, the negative standard logarithm of the adjusted *p* value (−log[FDR]) is plotted.

To identify the differences between the *in vitro* models of human skeletal muscle cells and their *in vivo* counterparts, we assembled a comprehensive dataset of more than 400 samples from 34 studies with bulk RNA-seq and 5 with sc- or snRNA-seq. Our analyses included hPSCs, hPSC-derived myogenic progenitors (DMPs), 2D and 3D hPSC-derived myotube cultures (DMTs), fibroblasts (FBs), fibroblast-derived transdifferentiated myoblasts and myotubes (TDMBs and TDMTs), adult tissue-derived myogenic progenitors (AMPs), fetal and embryonic myogenic progenitors (FMPs and EMPs), adult isolated myofibers (AMFs), heterogeneous adult and fetal biopsies (FSMs), immortalized myogenic cell lines (iMB and iMT), and primary cultures of human muscle cells including myoblasts and myotubes (pMBs and pMTs) (Figure 1A).

A principal-component analysis (PCA) of all bulk RNA-seq samples demonstrated a clear divide between the *in vitro* models and the *in vivo* reference samples, with the adult samples separating from the others and the *in vitro* samples positioning closer to FSMs (Figure 1B). Additionally, performing a correlation matrix between all included cell types revealed that both the differentiation and transdifferentiation models are more closely correlated with fetal muscle (r = 0.66 and r = 0.56) than adult muscle (r = 0.34, r = 0.2) (Figure S1A). To exclude possible technical biases due to the integration of large number of samples, we highlighted that within the different models, multiple samples from different laboratories contribute to their respective model clusters (Figure S1B). To interrogate potential technical biases in more detail, we investigated the separation between the hPSC and DMT samples of 5 individual laboratories, showing that the major separation can be found between these two major cell types (Figures S1C and S1D), although a smaller fraction of separation could be attributed to technical differences between laboratories. We then explored the individual contributors to the variance between samples by performing a principal variance component analysis (PVCA) (Figure S1E). We included 6 variables, both biological and technical: (1) cell type, (2) sequencing platform, (3) differentiation protocol based on major differences in media supplementation, (4) laboratory of origin, (5) the type of hPSCs, and (6) culture substrate. This analysis demonstrates that the largest variance between samples can be explained by the biological variable, cell type. However, the technical effects, such as the sequencing platform also explain a part of sample variance. To minimize such technical biases and to ensure that the differential expression of the genes we investigated in downstream analyses result from biological effects, we have applied strict dual filtering criteria. For upregulated genes, the median counts per million (CPM) per gene was to exceed 5 (CPM > 5), while for downregulated genes the median CPM was set to be below 1 (CPM < 1). To check the distribution of gene expression levels within sample groups, we applied an empirical cumulative distribution function. Nearly 90% of all 7,272 filtered genes passed the requirements for both filters across the majority of replicates of each sample type (CPM <1 = 87.71% and CPM >5 = 86.28%), showing high levels of homogeneity in gene expression across replicates derived from different laboratories and protocols (Figures S1F and S1G). This consistent pattern across replicates supports the notion that the highlighted differences between cell groups are caused by biological effects rather than technical biases. Finally, a group of the included samples, such as those derived from biopsies, are composed of heterogeneous cell populations despite being mainly myogenic. Our filtering strategy for the downregulated genes also addresses the challenge of analyzing such samples by selecting the genes, whose expression is completely absent across all cell types within a heterogeneous population, ruling out the possibility of detecting expression due to the presence of non-myogenic cell types.

We then investigated the expression levels of myogenic regulatory factors (MRFs), which are transcription factors that have master regulatory roles during the highly orchestrated process of myogenesis. The main MRFs include *MYF5*, *MYOD1*, *MRF4* (*MYF6*), and *MYOG*, and their temporal expression is important for the successful completion of muscle development and regeneration (Asfour et al., 2018). Additionally, we investigated the expression of *PAX7*, a marker of early myogenesis and resident muscle stem cells, called satellite cells. During the myogenic progenitor stage, there is clear upregulation of *MYF5* and *PAX7* in the DMP and the AMP, although the expression of these myogenic markers is virtually absent in the transdifferentiation model (Figure 1C). *MYOD1* marks the transition of myogenic progenitors toward myoblasts and primes them for myotube differentiation. As expected, this MRF is highly upregulated in the transdifferentiation model owing to the commonly used method of *MYOD1* overexpression. *MYOD1* levels in hPSC-based differentiation model, however, were similar to adult and fetal muscle samples (Figure S1H). *MYOG* expression is present in the mature stage of both *in vitro* models and the *in vivo* references, whereas *MYF6* failed to be expressed specifically in the transdifferentiation model (Figure 1C).

To get a first insight into the main differences between DMT and TDMT in comparison to AMF, we examined the overlap in downregulated differentially expressed genes (DEGs) between the two models. As myotube cultures are typically heterogeneous, we focused on downregulated DEGs with strict expression criteria (CPM <1 *in vitro*) to identify the genes that are virtually absent *in vitro* as opposed to *in vivo*. Our dataset includes several studies with different *in vitro* protocols, which further consolidates the confidence in the commonly downregulated genes across these protocols. In total, DMT and TDMT cultures showed an overlap of 570 DEGs, highlighting a striking overlap of more than 60% of their total number of DEGs (Figure 1D). Predicted protein-protein interactions using STRING revealed four groups of genes that are predicted to be interacting highly as an interconnected group (Figure 1E, left). Enriched gene ontology terms for these four major gene groups suggested a function for these genes in striated muscle contraction and structure (*MYH1*, *MYL2*, and *TCAP*), lipid metabolism (*FMO2*, *LPL*, and *PPARG*), and surprisingly also endothelial cells (*ANGPT2*, *SOX7*, and *KANK3*) and the immune system (*RORC*, *IL18*, and *IL16*) (Figure 1E, right).

To rule out the possibility of a major contamination of immune and endothelial cells in the isolated AMF, we analyzed a recent snRNA-seq dataset of a complete adult muscle biopsy for the expression of the genes predicted to be related to these cell types (Pass et al., 2023) (Figure S2A). Twenty-five percent of the genes enriched in the gene ontology terms related to these cell types were simultaneously expressed in the myofibers. Ten of these genes were robustly expressed at high levels in the myofiber-associated nuclei (Figures S2B–S2K), suggesting previously uncharacterized functions for these genes within adult myofibers. Twenty additional genes showed low to medium expression within myofiber-associated nuclei (Figure S2L). In summary, MRFs and genes that fail to be expressed in both *in vitro* systems show that *in vitro* models exhibit disparities compared to the *in vivo* references, although they resemble the fetal stages more. They also lack the expression of structural and lipid metabolism-related genes associated with adult skeletal muscle in human and a group of previously overlooked genes.

### Aberrant expression of transcription factors and epigenetic complexes in the *in vitro* models

**Figure 2:**
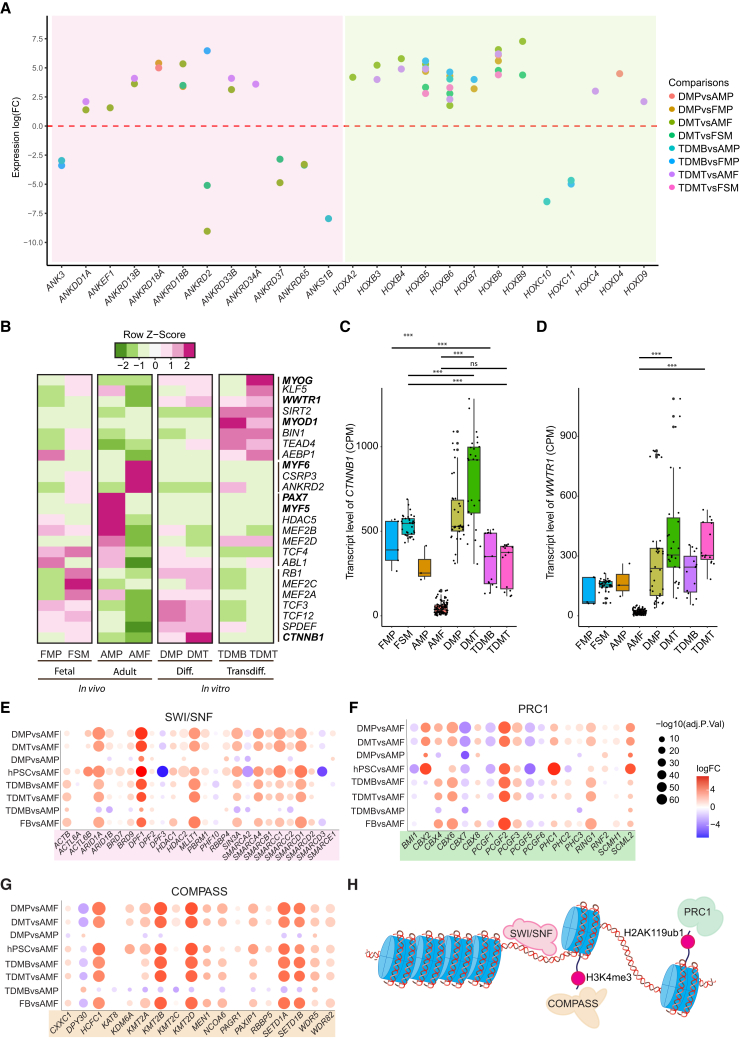
Analysis of expression of transcription factors and epigenetic complexes in the *in vitro* models (A) Dot plot showing the positive and negative standard logarithmic fold change of gene expression for DEGs across the indicated comparisons. Each unique comparison is color-coded, and genes are grouped on the *x* axis based on the transcription factor families they belong to, with the ANKRD family being on the left and HOX family on the right. (B) Heatmap demonstrating the average CPM values for transcription factors associated with the myogenic identity. (C and D) Boxplots showing expression levels of *CTNNB1* and *WWTR1*, across all categories (Student’s *t* test, *p* < 0.05). Data are presented as the mean ± SE. (E–G) Dot plot demonstrating the expression of the active members of the SWI/SNF (E), PRC1 (F), and COMPASS/MLL (G) complexes across three stages of hPSC-derived differentiation and fibroblast-derived transdifferentiation models. Source material, which are hPSCs and fibroblasts, and *in vitro* generated myotube samples were compared to AMF, whereas the mononucleated myogenic intermediates were compared to AMP and AMF. Dot colors indicate standard logarithmic fold change and dot size is determined by the negative standard logarithm of the adjusted *p* values for the respective fold change. (H) Schematics illustrating the canonical chromatin function of the epigenetic complexes highlighted in Figures 2E–2G.

Subsequently, we sought to investigate the expression of major drivers of cell fate changes, namely the transcription factors and epigenetic complexes, in the *in vitro* models compared to *in vivo*. DEGs were filtered through a comprehensive list of human transcription and epigenetic factors. First, multiple members of the HOX family of transcription factors were consistently upregulated in both myoblast and myotube stages of hPSC-derived differentiation and fibroblast-derived transdifferentiation models in comparison to isolated AMF (Figure 2A). The same trend was also recapitulated for several members of the HOXB cluster in iMT (Figure S3A). HOX genes have a well-described role in spatial patterning during development and control muscle diversity, likely regulating initial fate specification *in vitro* (Nayak et al., 2021). Additionally, we found significant differential expression of the members of ankyrin repeat and death domain containing (ANKRD) transcription factor family, of which two have been described to play important roles in skeletal muscle, namely *ANK3* and *ANKRD2* (Figure 2A) (Bean et al., 2014; Hopitzan et al., 2005).

We then focused on the expression patterns of the transcription factors that are associated with myogenic identity. β-catenin (*CTNNB1*), was highly expressed in hPSC-derived differentiation model, whereas it showed very low expression levels in AMF, suggesting the possibility of a sustained, aberrant Wnt activity in the *in vitro* system (Figures 2B and 2C). Importantly, the small molecule, CHIR99021, which activates the Wnt pathway through GSK3β inhibition, has been suggested to enhance the efficiency of transdifferentiation and is also commonly used in widely accepted hPSC-derived skeletal muscle differentiation protocols (Chal et al., 2016; Xu et al., 2020). Similarly, *WWTR1* showed higher expression levels in both *in vitro* models as compared to fetal or adult references (Figure 2D). Finally, we found that several active members of three major epigenetic complexes, SWI/SNF, PRC1, and COMPASS, had altered expression patterns in the *in vitro* models as compared to their references throughout the different stages of myogenesis starting from their source material (Figures 2E–2H). Both SWI/SNF and PRC1 have been shown to play pivotal roles in myogenesis and cell differentiation (Asp et al., 2011; Sharma et al., 2021). Therefore, dysregulation of their active members might affect the efficiency of generation of *in vitro* skeletal muscle as retention of the epigenetic memory from the source cells might form a roadblock for conversion (Figures S3B–S3D).

### Dysregulation of metabolic homeostasis and fiber type signatures of *in vitro* models

**Figure 3:**
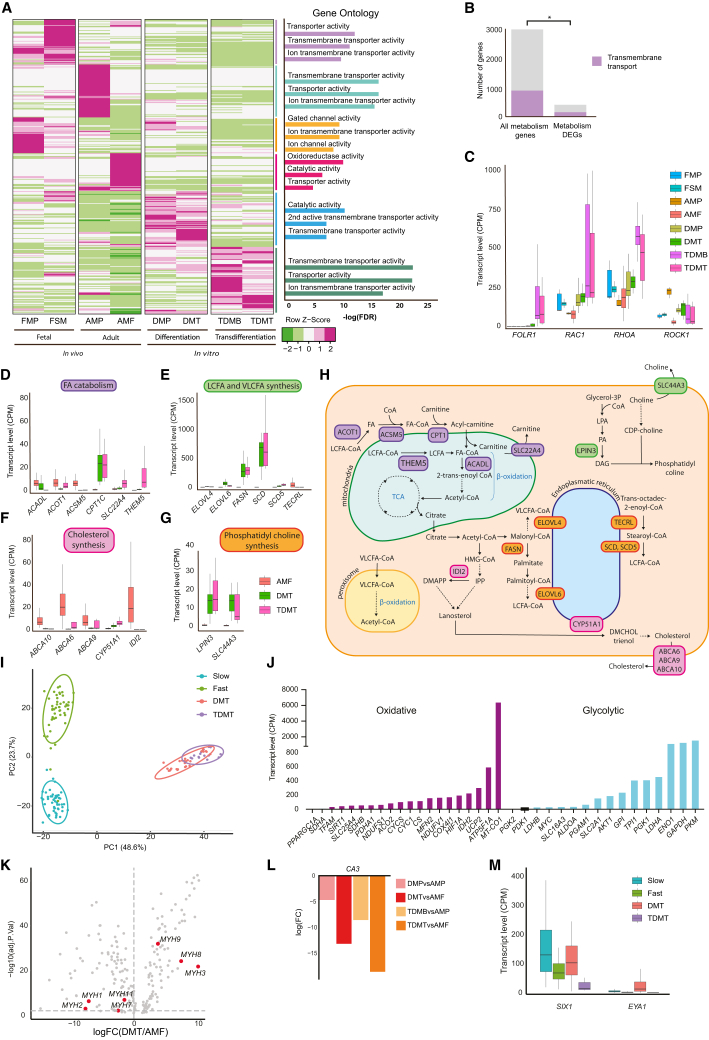
Metabolism and fiber type signatures of *in vitro* models of human skeletal muscle (A) Heatmap showing metabolism DEGs in the *in vitro* models, plotted as transformed *Z* score for the average CPM across all categories. DEG blocks characteristic for each sample are analyzed individually using GSEA and the enriched gene ontologies are summarized in the bar plot on the right. (B) Bar plot highlighting the significant enrichment (*p* = 0.00016, proportion test) of the proportion of genes associated with transmembrane transport within the differentially expressed metabolism genes as compared to their proportion within all metabolism genes. (C) Bar plot of average expression levels (CPM) of members of folic acid cycle across *in vivo* and *in vitro* samples. (D–G) Bar plots highlighting average expression levels (CPM) of members of major subprocesses of fatty acid and lipid metabolism, including fatty acid catabolism (D), long chain fatty acid and very-long-chain fatty acid synthesis (E), cholesterol synthesis (F), and phosphatidyl choline synthesis (G). (H) Schematics illustrating lipid and fatty acid cycles and the roles of the genes highlighted in Figures 3D–3G within each respective subprocess. (I) PCA of isolated human skeletal muscle fiber type 1 and 2 samples compared to TDMT and DMT, based on fiber type-specific marker genes. (J) Bar plot showing average expression levels (CPM) of genes implicated in glycolytic or oxidative energy metabolism for the hPSC-derived differentiated myotubes. (K) Volcano plot showing the differentially expressed myogenic genes between the DMT and the AMF, highlighting different Myosin Heavy Chains. (L) Bar plot demonstrating the logarithmic fold change of *CA3* expression for DMP vs. AMP, DMT vs. AMF, TDMB vs. AMP, and TDMT vs. AMF. (M) Bar plot showing the transcript levels (CPM) of *EYA* and *SIX1* genes, for the tissue-isolated fiber types and *in vitro* DMT and TDMT. Data are presented as the mean ± SE.

The different stages of myogenesis are supported by specific changes in metabolism. Metabolic reprogramming is a major component of muscle differentiation as it switches progenitors from a quiescent to an active state in the adult stem cell population (Ryall, 2013). To get insight into potential metabolic differences between the *in vitro* models and the *in vivo* references, we first investigated global changes of expression in metabolism-related genes. Across all metabolism-related genes, transmembrane transporters were found as a significantly enriched group that was differentially expressed across all stages of *in vitro* models (Figures 3A and 3B). Interestingly, metabolism-related DEGs were also enriched within gene ontology terms related to folic acid metabolism. Indeed, both the folic acid receptor *FOLR1* and three other active members of the pathway, *RAC1*, *RHOA*, and *ROCK1*, were significantly upregulated in the hPSC-derived differentiation model, while they showed a more striking upregulation in the transdifferentiation model (Figure 3C). In addition, members of several phases of the lipid cycle, including fatty acid catabolism, long and very-long-chain fatty acid synthesis. and phosphatidyl choline synthesis, were upregulated at the DMT and TDMT stages in comparison to the isolated AMF (Figures 3D, 3E, 3G, and 3H). Conversely, expression of members of the cholesterol synthesis pathway was downregulated in both *in vitro* models of human multinucleated myotubes (Figures 3F and 3H). Members of folic acid metabolism and lipid cycle were also upregulated in iMT as compared to AMF, while members of cholesterol synthesis pathway were downregulated (Figures S4A and S4B–S4E). These observations suggest a dysregulation in folic acid and lipid metabolism across different models.

Protein homeostasis is another tightly regulated skeletal muscle process. The ankyrin repeat and SOCS Box (ASB) gene family encodes subunits of the E3 ubiquitin ligase complex, which has critical functions in protein turnover. Seven members of the ASB family were entirely absent from the *in vitro* models at both MB and MT stages as compared to either fetal or adult *in vivo* references, highlighting major dysregulation in the protein homeostasis machinery in the *in vitro* models (Figure S4F).

Skeletal muscle fibers can differ in their preference of energy metabolism based on their fiber subtypes. We investigated if *in vitro* models generate a specific fiber subtype that resembles either oxidative slow-twitch fibers (type I) or glycolytic fast-twitch fibers (type II). A PCA based on the genes associated with fiber type signatures suggested that DMT and TDMT cultures differed equally from both slow and fast fiber types but not from each other (Figure 3I) (Chemello et al., 2019; Dos Santos et al., 2022; Zhang et al., 2024). For both *in vitro* models, genes related to oxidative and glycolytic metabolism were upregulated, although a larger fraction of glycolysis-related genes had high expression levels, potentially suggesting that these cultures may be more glycolytic (Figures 3J, S4I, and S4J).

Myosin heavy chains play an important role in muscle contraction. *MYH1*, *MYH2*, and *MYH4* have been identified to be mainly expressed in fast type muscle fibers, whereas *MYH6* and MYH7 are associated with the slow fiber type (Stuart et al., 2016). Although several of the fast fiber type-related myosin heavy chains were upregulated in the *in vitro* systems, another group of them were downregulated (Figures 3K and S4G). Additionally, between TDMT and DMT, DMT had higher expression of *MYH7*, *MYH4*, and *MYH3* (Figure S4H). The expression of *CA3*, a slow fiber-associated carbonic anhydrase, was drastically downregulated in both *in vitro* models, supporting the notion of a more fast fiber-like phenotype (Figure 3L) (Huang et al., 2019a).

Finally, we analyzed the expression of the bipartite transcription factor complex *EYA1*-*SIX1*, which has been shown to promote the reprogramming of slow-twitch fibers to fast-twitch type (Grifone et al., 2004). We show that the DMT have considerable levels of expression of both members of the complex, arguing for a fast fiber type signature (Figure 3M). These analyses suggest that both hPSC-derived differentiation and fibroblast-derived transdifferentiation models recapitulate some aspects of the fast-twitch fiber phenotypes, although their fiber type identity does not strictly adhere to one single type.

### Altered landscape of signaling pathways in models of skeletal muscle

**Figure 4:**
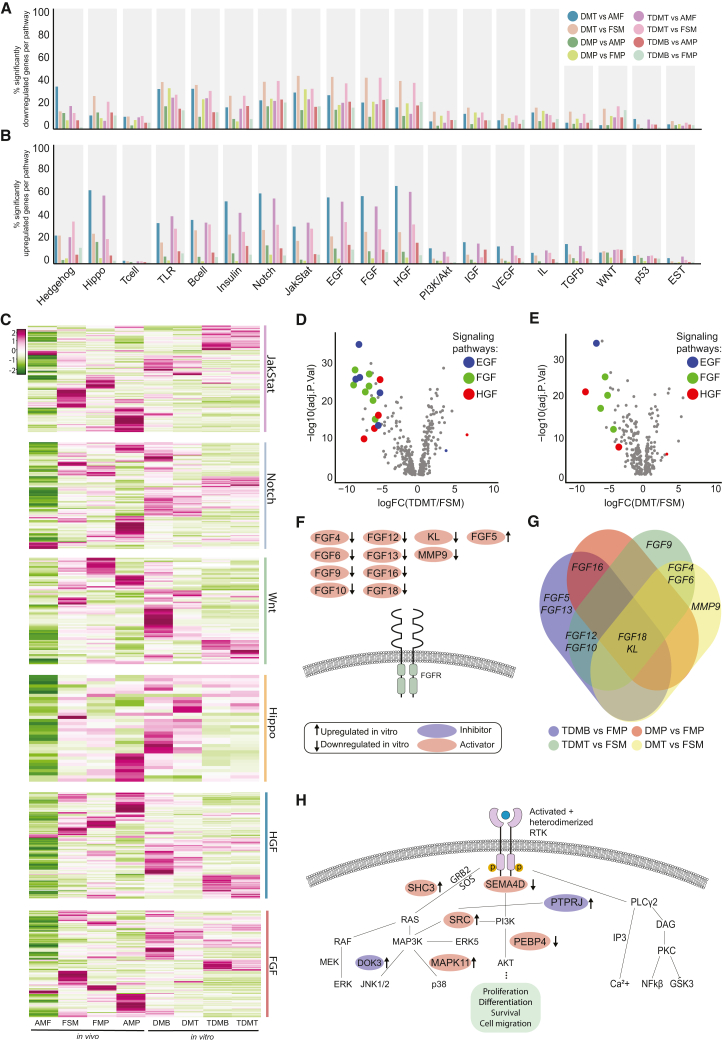
Signaling pathway landscape of *in vitro* models of human skeletal muscle (A and B) Bar plots showing percentages of significantly downregulated (A) and upregulated (B) members of individual signaling pathways across all indicated comparisons. (C) Heatmap showing average transcript levels (CPM) of all members of six highlighted signaling pathways in the *in vivo* and *in vitro* samples. (D and E) Volcano plots of DGEs in TDMT (D) and DMT (E) in comparison to FSM. Significantly downregulated members of the EGF (blue), HGF (red) and FGF (green) signaling pathways are highlighted in color. (F) Schematics depicting all significantly downregulated (FDR < 0.05) ligands of the FGF signaling pathway for transdifferentiation and hPSC-derived differentiation models at MB and MT stages compared to fetal references. (G) Venn diagram displaying the individual comparisons that revealed the genes summarized in (F), highlighting overlapping FGF ligands across multiple comparisons. (H) Schematics depicting significantly up- and downregulated members of downstream receptor tyrosine kinase signaling for TDMT vs. AMF. Red label indicates activators of the pathway, while blue label shows the inhibitors. Up- and downregulated members are indicated by arrows in either direction, respectively.

The precise expression of specific signaling pathways is tightly regulated to ensure the successful progression of cell fate commitment during development. In the context of developmental and postnatal myogenesis, signaling pathways such as Wnt, Notch, Sonic hedgehog, and fibroblast growth factor (FGF) have been shown to play indispensable roles (Chal and Pourquié, 2017). Supplementation of small molecules to alter or boost signaling pathways in the *in vitro* models has been described to have beneficial effects on the size and maturation of skeletal muscle cells (Shahriyari et al., 2022). We compiled a comprehensive gene set of 19 pathways with well-established and lesser-known functions in myogenesis. First, we interrogated the different patterns of significantly up- and downregulated members of individual signaling pathways. We observed the highest percentage of downregulated pathway members (40%) for the insulin growth factor (IGF), Notch, Janus kinase/signal transducer and activator of transcription (JAK/STAT), epidermal growth factor (EGF), FGF, hepatocyte growth factor (HGF), the Toll-like receptor (TLR), and B cell pathways (Figure 4A). All these pathways, together with the Hippo pathway, also had up to 60% of their members significantly upregulated *in vitro* compared to *in vivo* (Figure 4B). To reveal the largest discrepancies between the signaling landscape of *in vitro* models and *in vivo* references, we utilized stringent expression cutoffs for up- and downregulated signaling pathway members. This analysis showed the Hippo, Notch, FGF, and HGF pathways as the most divergent ones in comparison to the adult myofibers and Notch, JAK/STAT, FGF, and Wnt pathways when compared to fetal samples (Figures S5A and S5B). Importantly, AMF differed drastically from all stages of *in vitro* models, mainly due to low expression of these pathway members (Figure 4C). A correlation matrix for the different sample groups based on these signaling pathways confirmed a higher correlation between the *in vitro* models and fetal muscle samples (Figure S5C). It has been shown that fetal and adult skeletal muscle have different transcriptomic profiles as fetal skeletal muscle includes more proliferating cells (Xi et al., 2020). Although the *in vitro* samples are known to have a profile that is more akin to that of fetal skeletal muscle, we observed substantial downregulation of various members of the EGF, FGF, and HGF pathways in the TDMT and DMT compared to FSM, suggesting that they do not also fully model fetal skeletal muscle at the current state-of-the-art (Figures 4D and 4E). Since FGF ligands were most commonly downregulated, we explored the individual deviations in FGF ligand expression for the different stages of *in vitro* muscle development compared to FMP and FSM. Ten FGF ligands were strictly missing in the *in vitro* models, whereas only one ligand, *FGF5*, showed upregulation (Figure 4F). However, the upregulation of *FGF5* was only observed in the TDMB compared to FMP, while it was not differentially expressed in other comparisons (Figure S5D). We also observed a group of ligands that were commonly missing in different pairwise comparisons (Figure 4G). *FGF18*, together with the FGF-activating enzyme KL, were missing in all stages of *in vitro* models compared to fetal samples. The absence of these ligands needed for the highly coordinated FGF signaling and its specific ligand-receptor interactions could exert a limiting effect on myogenesis. Indeed, FGF plays a regulatory role in mesoderm fate specification during early development and somite formation (Ciruna and Rossant, 2001) Finally, multiple genes related to downstream signaling cascades of receptor tyrosine kinases were identified among the DEGs of the transdifferentiation model compared to *in vivo* samples. These signaling cascades can lead to diverse outcomes, ranging from proliferation and differentiation to survival and cell migration. In TDMT compared to AMF, two activators of the PI3K/AKT pathway, *SEMA4D* and *PEBP4*, were downregulated, while MAPK/p38 pathway members, *SHC3* and *MAPK11*, were upregulated (Figure 4H). The p38 pathway is known to promote the *MYOD1* activity, suggesting that the increase in expression of the members of this pathway could be related to *MYOD1* activity during *MYOD1*-induced fibroblast transdifferentiation (Segalés et al., 2016). A comparison between the transdifferentiated cultures and fetal samples also demonstrated downregulation of *PLCB2*, which is an important activator of the phospholipase C and inositol triphosphate calcium (Ca^2+^) signaling cascade (Figure S5E). Ca^2+^ signaling plays a crucial role in the regulation of myoblast differentiation during development but also adult regeneration, suggesting that dysregulation of this pathway might contribute to an incomplete myogenic identity in the transdifferentiation model (Valdés et al., 2013).

**Figure 5:**
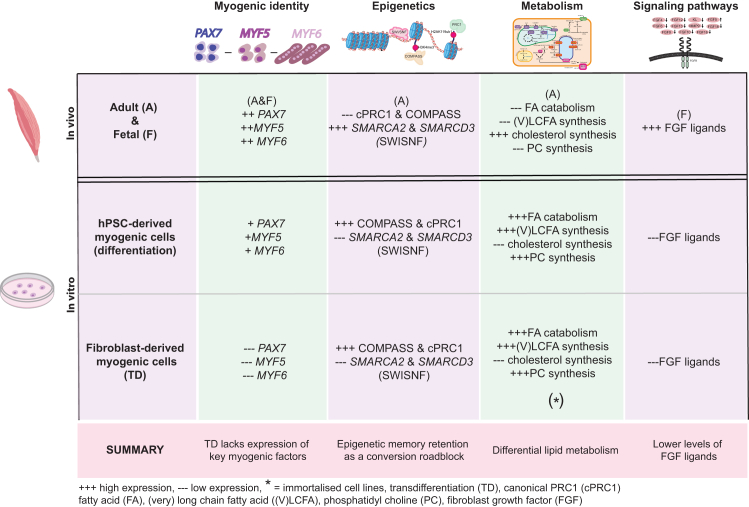
Summary of key findings of bulk RNA sequencing analyses of *in vitro* derived skeletal muscle models compared to *in vivo* references Summary table highlighting the key findings for 1–4 in the context of (1) myogenic identity, (2) epigenetics, (3) metabolism and (4) signaling pathways. Differences in expression are depicted by + and −, ranging from +++ for highest expression and −−− for lowest expression.

Our analysis upon integration of over 400 bulk RNA-seq samples led to several key findings that suggest (1) dysregulation of myogenic identity in the transdifferentiation model, (2) retention of the epigenetic memory in the *in vitro* models, (3) differential lipid metabolism between *in vitro* models and *in vivo* references, and (4) lower levels of FGF ligands in the *in vitro* models compared fetal skeletal muscle (Figure 5).

### Differences in transcriptomic identity of PAX7^+^ satellite cells *in vitro* and *in vivo*

Finally, we aimed to characterize *PAX7*^+^ populations in the *in vitro* models in comparison to their *in vivo* references of human skeletal muscle at different stages of life. *In vivo*, *PAX7* expression denotes 2 different stages of myogenic progenitor cells. The first one is a group of developmental, proliferating progenitors, which are myogenically committed to eventually fuse and form myofibers during embryonic development. The second group consists of set-aside, quiescent stem cells, also termed satellite cells or resident muscle stem cells, with an important role in adult muscle regeneration. Satellite cells have been proven hard to study *in vitro* since they quickly lose their quiescent nature after biopsy and isolation procedures (Van Den Brink et al., 2017). Therefore, research has been dedicated to recapitulating the satellite cell phenotype *in vitro* to be able to investigate this important cell type.

**Figure 6:**
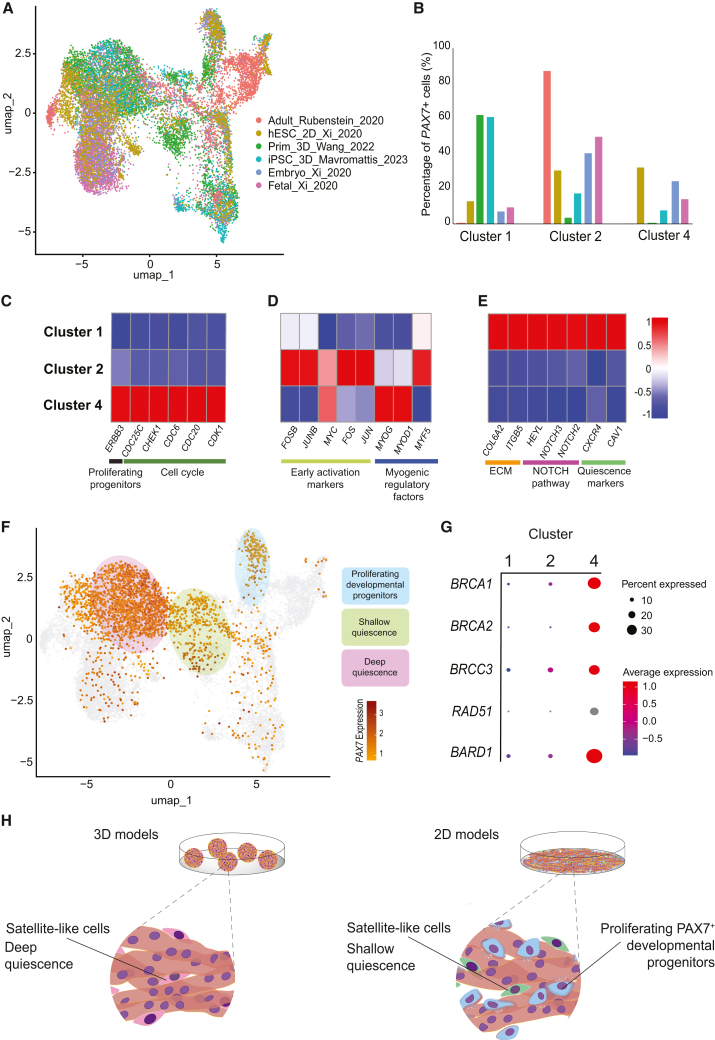
Differences between distinct populations of *PAX7*^+^ myogenic cells generated in 2D and 3D models of human skeletal muscle (A) UMAP showing the integrated scRNA-seq studies. The cellular origins of each *in vitro* model or the developmental stage of *in vivo* samples are indicated at the beginning of each sample label, followed by the mode of culture model (2D vs. 3D), last name of the first author of the study and the year of publication. (Prim, primary myogenic cells derived from biopsies). (B) Bar plot displaying the fraction of *PAX7*^+^ cells in the indicated clusters within the total number of *PAX7*^+^ cells in each study. The studies are color-coded as in Figure 5A. (C–E) Heatmaps highlighting the DEGs between the three indicated clusters; genes associated with proliferating developmental progenitors (C), genes related to a shallow quiescent state (D), and genes related to a deep quiescent state (E). (F) Annotation of the clusters of interest: deep quiescent cluster (1, pink), shallow quiescent and early activated satellite cell cluster (2, green), and proliferating developmental progenitor cluster (4, blue). (G) Dot plot demonstrating the level and the percentage of expression of genes associated with the BRCA1-BRCA2-containing Complex within indicated clusters. (H) Model highlighting the generation of satellite-like cells at different quiescent states from 2D and 3D differentiation methodologies.

To interrogate the differences of 2D and 3D *in vitro* primary cell- and hPSC-derived differentiation models in comparison to different developmental stages of human skeletal muscle, we analyzed single cell transcriptomics datasets. Surprisingly, majority of the limited available single cell transcriptomics datasets are derived from scRNA-seq, although the largest fraction of skeletal muscle or its *in vitro* models is composed of multinucleated myofibers or myotubes, respectively. scRNA-seq technology can mainly capture mononucleated cells and as a consequence of this incompatibility, myofiber/myotube-associated transcriptomes are largely underrepresented in these analyses, whereas snRNA-seq can more faithfully capture the dominant representation of the myofibers within skeletal muscle (Figures S1A vs. S6A). Based on this comparison, we strongly argue for the use of snRNA-seq, when myofiber-associated transcriptomes are studied. Nevertheless, for the interrogation of mononucleated PAX7^+^ skeletal muscle progenitors or stem cells, scRNA-seq datasets provide a highly valuable platform. Thus, we integrated six publicly available scRNA-seq datasets (Figure 6A), covering 16,582 cells (Figure S6B). By performing clustering following the Louvain algorithm, an appropriate resolution for the integrated dataset was set at 0.5 (Figures S6C and S6D). In total, 15 clusters were identified across these 6 datasets (Figure S6E). The clusters were annotated based on a set of marker genes that was built by compiling the individual gene sets from the datasets (Figure S6F). All individual clusters were assessed for the percentage of *PAX7* expressing cells and the level of average *PAX7* expression per study and per cluster. Based on these criteria, three clusters were found to be the most prominent *PAX7*^+^ clusters (Figures S7A and S7B). Interestingly, the *PAX7*^+^ population in cluster 1 was mainly composed of cells derived in 3D organoid studies, with smaller contributions from the other samples. Cluster 2, on the other hand, was the only cluster with *PAX7*^+^ cells identified in adult human skeletal muscle. Finally, cluster 4 consisted mainly of *PAX7*^+^ cells identified in fetal and embryonic muscle and 2D hPSC-derived differentiation model (Figure 6B). Despite having a small percentage of *PAX7*^+^ cells, cluster 0 showed low average *PAX7* expression in comparison to the other clusters and was identified as mesenchymal stem cells based on the robust expression of a large group of markers (Figure S6F). We next performed pairwise differential gene expression analysis between the clusters to identify unique cell populations, while also revealing marker genes for each of these three clusters. This analysis showed high upregulation of cell cycle-related genes and the proliferative myogenic progenitor marker *ERBB3* in cluster 4 compared to the other two clusters (Figure 6C) (Figeac et al., 2014). This proliferative phenotype was supported by the presence of other cell cycle-related genes, such as *CDC7* and *CCNB1*, among the top 20 cluster identifier marker genes (Figure S7C). Cluster 2, instead, showed upregulation of early satellite cell activation markers, *MYC*, *FOS*, and *JUN*, and had the highest *MYF5* expression (Figure S7E). *MYOG* and *MYOD1* were expressed at low levels in clusters 1 and 2 but were robustly upregulated in cluster 4 (Figures 6D and S7D). Finally, cluster 1 was characterized by the differential expression of several extracellular matrix proteins, Notch signaling pathway members and two markers of satellite cell quiescence, *CXCR4* and *CAV1* (Figure 6E) (Sherwood et al., 2004; Volonte et al., 2005). Based on these characterizations, we conclude that the *PAX7*^+^ cells in cluster 4 are mainly proliferating, EMP, while those in clusters 1 and 2 are deep and shallow quiescent satellite cells, respectively, revealing a continuum of quiescence across models (Figure 6F).

Surprisingly, we also observed a significant enrichment of expression of five members of the BRCA1-BRCA2-containing complex (BRCC) in cluster 4, specifically *BRCA1*, *BRCA2*, *BRCC3*, *RAD51*, and *BARD1* (Figure 6G). There is limited evidence of the role of BRCC in myogenesis; however, it has been shown that one of the members of BRCC, *BABAM2*, enhanced the differentiation and fusion of adult satellite cells in mouse skeletal muscle regeneration (Xiao and Lee, 2016).

Thus, our analyses reveal that the 2D hPSC-derived differentiation model generates a small subset of deep quiescent satellite-like cells and a larger group of shallow quiescent satellite-like cells with a similar sized group of proliferating developmental progenitors. Conversely, 3D skeletal muscle organoids, regardless of whether they were derived from hPSCs or primary cells, mainly give rise to satellite-like cells with a deep quiescence signature (Figure 6H).

## Discussion

*In vitro* models are pivotal to study development, disease, and general biological processes. The objective of this study was to discern discrepancies between the current skeletal muscle models and adult, fetal, and embryonic human skeletal muscle to reveal the cellular processes that can be targeted to improve the current models. We highlighted disparities in different processes such as myogenesis, transcription and epigenetic factor expression, metabolism, signaling, and progenitor generation. Such an extensive meta-analysis of published bulk RNA-seq and scRNA-seq datasets derived from several different *in vitro* models has not been performed to date for human cells. Thus, this study provides novel insights on the missing elements in human skeletal muscle models.

Our analyses revealed that early and late-stage MRFs *MYF5* and *MYF6* are missing in the transdifferentiation model. Although the absence of *MYF5* can be explained by the possibility that the transdifferentiation happens without passing through a progenitor state, the lack of *MYF6* expression indicates an incomplete differentiation transcriptome in this model as *MYF6* was shown to be essential for the expression of a set of genes important for skeletal muscle maturation (Hernández-Hernández et al., 2017; Mak et al., 1992). *MYOD1* overexpression has been shown to incompletely convert fibroblasts into muscle cells regarding gene expression, myotube stability and DNA methylation profiles in mouse embryonic fibroblasts (MEFs) and a four-day transdifferentiation of human fibroblasts (Cacchiarelli et al., 2018; Radwan et al., 2024; Yagi et al., 2021). Co-overexpression of *MYF6* and *MYOD1* could therefore be used to enhance transdifferentiation.

We showed that the *in vitro* models had aberrant expression of genes related to lipid metabolism and the fatty acid cycle. Lipid metabolism plays an extensive role in skeletal muscle as it is one of its main energy sources. Aberrant upregulation of genes within several lipid metabolism pathways could be caused by a failure in metabolic reprogramming, in particular in the case of the hPSC-derived differentiation model. *In vivo*, skeletal muscle is dependent on fatty acid uptake from the environment as it lacks the expression of fatty acid synthase (*FASN*) (Tanosaki et al., 2020; Thomson and Winder, 2009). The high expression of *FASN* in the *in vitro* models could be explained by the nutrient availability and the culture media composition. It was shown that hPSC metabolism is dependent on the nutrients that are available and changes in culture conditions could alter the metabolic pathways significantly (Zhang et al., 2016). Therefore, further optimization of the media composition for all *in vitro* models could enhance their metabolic similarities to bona fide muscle.

The ASB genes were consistently identified as missing in the *in vitro* models compared to *in vivo* references. The ASB gene family encodes subunits of the E3 ubiquitin ligase complex and most of its family members have been shown to have high expression in muscle (Ehrlich et al., 2020). One of the ASB genes, *ASB15* has been shown to promote muscle differentiation by regulating protein turnover (McDaneld et al., 2006).

Our analyses revealed that 8 FGF ligands were completely missing (Figure 4F) in the *in vitro* models. The role of FGF signaling in myogenesis has been rather controversial. FGF has been shown to both positively and negatively affect muscle differentiation in multiple model organisms and in *in vitro* culture (Armand et al., 2005; Huang et al., 2019b; Vishal et al., 2020). Additionally, *FGF2* is already an important component of hPSC-derived muscle differentiation protocols (Chal et al., 2016). Since the individual ligands likely have different effects, the addition and incubation time of FGF ligands in the culture media should be extensively tested.

Cell fate changes are accompanied by major alterations in the epigenetic landscape. The epigenetic memory of the source cells can be retained in experimental models, and in the case of fibroblast transdifferentiation toward the myogenic lineage, this has been shown to have an inhibitory effect (Manandhar et al., 2017). Our analysis highlighted two major dysregulated transcription factor families, HOX and the downregulation of ANKRD genes, of which only one has been described within a myogenic context before (Raymond et al., 2010). The aberrant gene expression patterns of these families could potentially be caused by differential developmental timing of *in vitro* models compared to the *in vivo* references.

We additionally showed that canonical PRC1 components (*BMI1*, *CBX6*, *PCGF2*, *PHC2*, *RING1*, and *SCMH1*) were upregulated in the *in vitro* models and this high expression was already present in their source cells, namely the hPSCs and fibroblasts, and failed to be erased upon differentiation or transdifferentiation, respectively. PRC1 stabilizes the expression of cell fate commitment genes. Therefore, chemical inhibition of the PRC1 complex could potentially enhance transdifferentiation by allowing the activation of muscle differentiation-specific gene networks.

Another major epigenetic complex that plays an important role in muscle differentiation is the SWI/SNF ATP-dependent chromatin remodeling complex with SMARCA2 and SMARCA4 being its main facilitators (De la Serna et al., 2001). These two SWI/SNF enzymes have distinct functions in myogenesis and are indispensable for the myogenic program. *SMARCA4* activates muscle gene transcription at the earlier stages of myogenesis while *SMARCA2* causes proliferating myoblasts to exit the cell cycle by repression of cyclin D1. Specifically, absence of *SMARCA2* expression in all stages of differentiation and transdifferentiation suggests that the cells may not fully exit the cell cycle, which in turn would limit their fusion capacity.

Finally, we analyzed an integrated dataset of six different scRNA-seq samples, covering four studies to identify transcriptomic identities of *PAX7*^+^ cells in the *in vitro* models. In the cluster containing the proliferating developmental *PAX7*^+^ progenitors, we identified the differential expression of the BRCA1-BRCA2-containing complex (BRCC), which encompasses *BRCA1*, *BRCA2*, and *BRCC3* in addition to the cell cycle genes. The BRCC complex has been described to play a role in skeletal muscle metabolism (Tarpey et al., 2021). Due to its high expression in the differentiating cluster, we hypothesize that this complex likely plays an important role during myogenic commitment in embryogenesis.

Interestingly, we observed that the 3D organoid models mainly generated satellite-like cells with a deep quiescence phenotype, marked by the expression of the NOTCH pathway and extracellular matrix genes (Tao et al., 2023). Conversely, hPSC-derived 2D differentiation model generated (1) a population of satellite-like cells at a shallow quiescence state, clustering together with the adult *PAX7*^+^ cells, (2) proliferating developmental *PAX7*^+^ progenitors, clustering together with the majority of the fetal and embryonic *PAX7*^+^ cells, and (3) a very small group of satellite-like cells with a deep quiescence phenotype. Therefore, we argue that 2D cultures give rise to a diverse *PAX7*^+^ cell population, while 3D cultures mainly produce *PAX7*^+^ satellite-like cells that show a deeper quiescence profile, potentially due to the enhanced extracellular matrix niche within the model.

Surprisingly, although a similar approach of cell isolation was used to dissociate the cells from the adult muscle and the 3D organoids, the adult muscle samples did not include any cells that showed a deep quiescent profile. We speculated that this might be due to the initial insult of the biopsy process, setting off an early activation response of satellite cells *in vivo*. The accumulation of more adult scRNA-seq studies could reveal a *PAX7*^+^ population in adult muscle, composed of deeply quiescent satellite cells. Since we included skeletal muscle biopsies within our bulk RNA-seq analysis, we do acknowledge cell heterogeneity as a limitation of our study. However, we have taken several measures to minimize any potential effects, including the use of isolated adult myofiber samples and strict CPM filtering criteria.

In conclusion, this large-scale meta-analysis covering more than 400 published bulk RNA-seq and seven scRNA-seq or snRNA-seq datasets, uncovered differences between *in vitro* human skeletal muscle models and *in vivo* references. Systematic characterization of these differences provides novel insights regarding skeletal muscle cell identity, while also suggesting adjustments in the protocols to improve the existing models. This large-scale dataset will also prove useful for future analyses employing unbiased pattern detection methods to identify unknown gene networks important for skeletal muscle development and function. This holistic approach can also be used for other cell types in human, paving the way for a better understanding of cellular identities and existing models.

## Methods

### Curation of the dataset

To perform the large-scale analysis of *in vitro* models of human skeletal muscle in comparison to *in vivo* samples, a thorough literature search led to a collection of a total of 418 bulk RNA-seq samples covering 34 independent studies (Data S1: SRA). For the *in vivo* references, only healthy control samples were included. FASTQ files for bulk RNA-seq samples and count matrices and metadata for sc- and snRNA-seq were downloaded from the Gene Expression Omnibus database (GEO; https://www.ncbi.nlm.nih.gov/geo/) and samples were labeled according to the study and cell type to facilitate identification. FastQC (version 0.12.0) was used for the quality control of all bulk RNA-seq data (Andrews, 2010).

### Preprocessing of data

A count table was generated by aligning the samples to the human reference genome (Gencode, release 44 [GRCh38.p14]) using STAR (version 2.5.26) and featureCounts (version 2.0.1). All samples were mapped using a PCA after normalization and logarithmic transformation. Additionally, a PVCA to quantify variance was performed using the variancePartition package in R. Following variables were included: (1) cell type (hPSC or DMT), (2) the sequencing platform used, (3) differentiation protocols based on major differences in media supplementation ([a] supplementation with DAPT, [b] with LDN193189 and FGF, and [c] no additional supplementation), (4) individual laboratories, (5) type of hPSC (hiPSCs or hESCs), and finally, (6) culture substrate (Matrigel or MEFs). Individual pairwise comparisons were selected from the master count table and raw gene counts, later converted to normalized CPM, were used as input for further analysis. Expression profiles from source cells for the *in vitro* generation methods (fibroblasts and hPSCs) were included as an initial point of comparison.

### Differential gene expression analysis and gene set curation

To identify the DEGs between the *in vitro* skeletal muscle models and their *in vivo* counterparts, the integrated Limma (version 3.5.1) (Ritchie et al., 2015) and EdgeR (version 4.0.16) (Robinson et al., 2009) workflow was used (Law et al., 2016). Only genes with an adjusted *p* value (false discovery rate [FDR]) <0.05 were used for further analysis. Significant DEGs were filtered for their gene expression levels: Downregulated genes had a median CPM expression of <1 for all samples of the group and therefore were virtually not expressed, while for the upregulated genes, a minimum median expression cutoff of CPM >5 was applied for all samples of the group. This resulted in a list of DEGs (up- and downregulated) per comparison. To identify functions and families of DEGs, lists were filtered for gene sets. Curated gene sets used included, myogenic genes (Croft et al., 2011; Liberzon et al., 2015), metabolic genes (Birsoy et al., 2015), transcription factors (Zhang et al., 2020), and epigenetic complexes (Marakulina et al., 2023). In addition, we compiled gene sets for a comprehensive list of signaling pathways, including Sonic hedgehog, Hippo, PI3K-Akt, T cell receptor, TLR, B cell receptor, insulin, Notch, JAK/STAT, EGF, IGF, VEGF, HGF, FGF, Akt-MTOR, interleukin, transforming growth factor β, Wnt, P53, and the estrogen signaling pathways (Rodchenkov et al., 2020).

### Downstream analysis of the differentially expressed genes

Gene ontology analysis was performed using the Gene Set Enrichment Analysis tool (GSEA; https://www.gsea-msigdb.org/gsea/) and the R tools MsigdbR (Subramanian et al., 2005) and clusterProfiler (Yu et al., 2012). In addition, STRING (Szklarczyk et al., 2019) version 12.0 (https://string-db.org/) was used to visualize the predicted protein-protein interaction networks.

### scRNA-seq: Preprocessing of datasets

scRNA-seq analysis was conducted using the Seurat package (version 5.0.2), beginning with the preprocessing of six individual datasets (Data S1: SRA). To prime the individual datasets for integration, a standardized method was followed (Hao et al., 2024).

### scRNA-seq: Dataset integration

FindIntegrationAnchors function was used to integrate the six datasets, aligning shared cell states across datasets. Batch effects and technical variations were corrected using the IntegrateData function, generating a unified integrated assay. The integrated dataset was scaled and reanalyzed using PCA. The Louvain algorithm was used to identify clusters after constructing a shared nearest neighbor graph. Optimal resolution was set at 0.5 resulting in 15 clusters visualized by uniform manifold approximation and projection (UMAP). Marker genes specific to each cluster were identified with FindAllMarkers, requiring a minimum log-fold change (logFC) of 0.25 and expression in at least 25% of cells within a cluster. To determine the clusters that are expressing *PAX7* highly, UMAP was applied to visualize the cells expressing *PAX7* in the clusters with a threshold of logFC >0.75.

### scRNA-seq: Differential gene expression analysis

To identify DEGs between the different *PAX7*^+^ clusters, LayerData was used to extract the count matrix from the integrated dataset and overlapping cells between the layer and specific metadata were identified. Subsets of *PAX7*^+^ cells were generated by filtering for cells with *PAX7* CPM >0. All subsets were merged into a single Seurat object for differential gene expression analysis, which was conducted using the FindMarkers function, applying a minimum logFC of 0.25 and requiring a minimum of 25% of cells to express the gene to identify genes differentially expressed between clusters.

### Statistics and reproducibility

For bulk RNA-seq and scRNA-seq datasets, DEG analyses were performed using EdgeR and Seurat, respectively. We have included a summary of all included samples, organized per category (Data S1: Samples). Differential expression was considered significant for FDR less than 0.05. Unless otherwise stated, the highlighted DEGs were filtered through significance for their respective comparisons. Figures 1C, 2C, 2D, S2B–S2D, 3C–3G, 3L, S3A, and S3C–S3F contain boxplots that demonstrate the average CPM expression per group and the standard error for the mean (SEM). Pairs of sample groups were compared to each other with a Student’s *t* test, and the differences were considered significant for *p* values <0.05. In Figure 3B, a proportion test was used to assess the significance of percentages.

## Resource availability

### Lead contact

Requests for further information and resources should be directed to and will be fulfilled by the lead contact, Atilgan Yilmaz (atilgan.yilmaz@kuleuven.be).

### Materials availability

This study did not generate new unique reagents.

### Data and code availability

All datasets used in this study are publicly available and can be downloaded from GEO or BioProject with the following accession codes: GSE129505, GSE121154, GSE111163, GSE87365, GSE161025, GSE234616, GSE221912, GSE178784, GSE93263, PRJNA610985, GSE158216, GSE214495, GSE236120, GSM1527072, GSE128844, GSE98509, GSE86356, GSE130646, GSE78158, GSE78644, GSE102812, GSE78649, GSE89588, GSE100943, GSE112101, GSE117609, GSE117382, GSE114938, GSE163213, GSE119402, GSE136807, GSE124072, GSE235781, GSE18927, GSE147513, GSE147514, GSE188215, GSE147457, and GSE130646. SRA numbers of individual samples can be found in Data S1 with their respective GEO accession number. Additionally, we have included the differentially expressed, CPM filtered genes for all comparisons in Data S1. Finally, codes and the raw integrated bulk RNA-seq count table can be downloaded from https://github.com/Atilgan-Yilmaz-Lab. All other data supporting the findings of this study and codes used for data analysis are available from the corresponding author.

## Acknowledgments

We thank A. Yildirim and M. Di Gloria for their assistance with data organization and gene set generation and B. van der Veer for his help in setting up the computing environment. This work was supported by the 10.13039/501100003130Research Foundation Flanders (FWO, Fonds voor Wetenschappelijk Onderzoek – Vlaanderen, G0DCO23N), Francqui Foundation (Francqui Stichting, ZKE2844/10338627) and KU Leuven (STG/22/042-ZKE3357). A.M.A. is supported by the 10.13039/501100003130FWO Doctoral Fellowship. A.Y. is Collen-Francqui Docent.

## Author contributions

M.V.P., A.M.A., E.E.M., J.B., and A.Y. designed the analyses. M.V.P., A.M.A., E.E.M., and J.B. curated and analyzed the data. M.V.P., A.M.A., E.E.M., J.B., S.J., and A.Y. interpreted the data. M.V.P. and A.Y. wrote the manuscript with input from all authors. A.Y. supervised the study.

## Declaration of interests

The authors declare no competing interests.

## Supplemental information

Document S1. Figures S1–S7

Data S1. Table including sample information (GEO and SRA) and differentially expressed gene lists from all individual comparisons

Document S2. Article plus supplemental information
