# Supplementary material for: Delineating transcriptomic signatures of in vitro human skeletal muscle models in comparison to in vivo references
Source: Stem Cell Reports. 2025 Oct 23;20(11):102684. doi: 10.1016/j.stemcr.2025.102684 (PMC12790736; doi:10.1016/j.stemcr.2025.102684)
Supplement: Document S1. Figures S1–S7 [file mmc1.pdf]

Stem Cell Reports, Volume *20*

Supplemental Information

Delineating transcriptomic signatures of *in vitro* human skeletal muscle models in comparison to *in vivo* references

Margaux Van Puyvelde, Eslam Essam Mohammed, Ángela Moreno Anguita, Jarne Bonroy, Sandra Jansen, and Atilgan Yilmaz

Supplemental information

Delineating transcriptomic signatures of in vitro human skeletal muscle models in comparison to in vivo references

Margaux van Puyvelde, Eslam Essam Mohammed, Ángela Moreno Anguita, Jarne Bonroy, Sandra Jansen, Atilgan Yilmaz

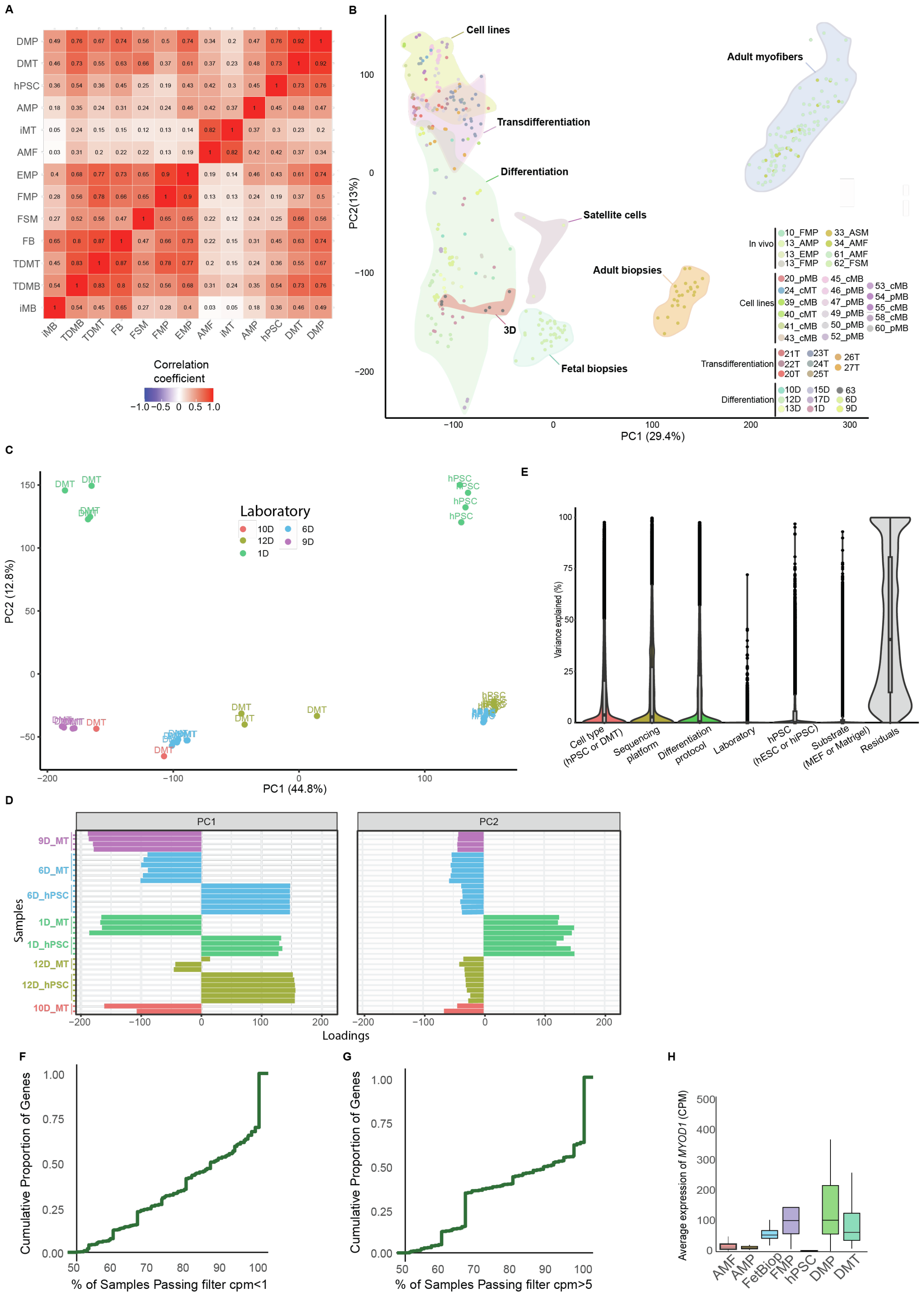
A
DMP
DMT
hPSC
AMP
iMT
AMF
EMP
FMP
FSM
FB
TDMT
TDMB
iMB
Correlation coefficient
−1.0 −0.5 0 0.5 1.0
B
Cell lines
Transdifferentiation
Differentiation
Satellite cells
Adult myofibers
Adult biopsies
3D
Fetal biopsies
PC2(13%)
PC1 (29.4%)
In vivo
10_FMP
13_AMP
13_EMP
13_FMP
33_ASM
34_AMF
61_AMF
62_FSM
Cell lines
20_pMB
24_cMT
39_cMB
40_cMT
41_cMB
43_cMB
45_cMB
46_pMB
47_pMB
49_pMB
50_pMB
52_pMB
53_cMB
54_pMB
55_cMB
58_cMB
60_pMB
Transdifferentiation
21T
22T
20T
23T
24T
25T
26T
27T
Differentiation
10D
12D
13D
15D
17D
1D
63
6D
9D
C
Laboratory
10D
12D
1D
6D
9D
PC2 (12.8%)
PC1 (44.8%)
E
Variance explained (%)
Cell type (hPSC or DMT)
Sequencing platform
Differentiation protocol
Laboratory
hPSC (hESC or hiPSC)
Substrate (MEF or Matrigel)
Residuals
D
PC1
PC2
Samples
9D_MT
6D_MT
6D_hPSC
1D_MT
1D_hPSC
12D_MT
12D_hPSC
10D_MT
Loadings
F
Cumulative Proportion of Genes
% of Samples Passing filter cpm<1
G
Cumulative Proportion of Genes
% of Samples Passing filter cpm>5
H
Average expression of MYOD1 (CPM)
AMF
AMP
FetBiop
FMP
hPSC
DMP
DMT

Figure S1 related to Figure 1: Correlation analysis between samples and assessment of replicate consistency. (A) Pearson correlation matrix of mean CPM values for all genes between all sample groups. Red signifies a positive correlation between groups, while blue highlights a negative correlation. (B) PCA plot of all samples, colored based on laboratory of origin. (C) PCA plot of hPSC and DMT samples colored by laboratory of origin. (D) PCA loading plot, which shows the individual samples and their directionality per principal component. (E) Principal Variance Component analysis (PVCA) highlighting the percentage of variance explained by six variables including cell type, sequencing platform, differentiation protocol, laboratory of origin, the type of human pluripotent stem cells (hPSCs) and culture substrate. (F-G) Empirical cumulative distribution function (ECDF) of all DEGs that were filtered with the CPM<1 filter (F) and the CPM >5 filter (G). (H) Bar plot showing mean expression (CPM) of *MYOD1*.

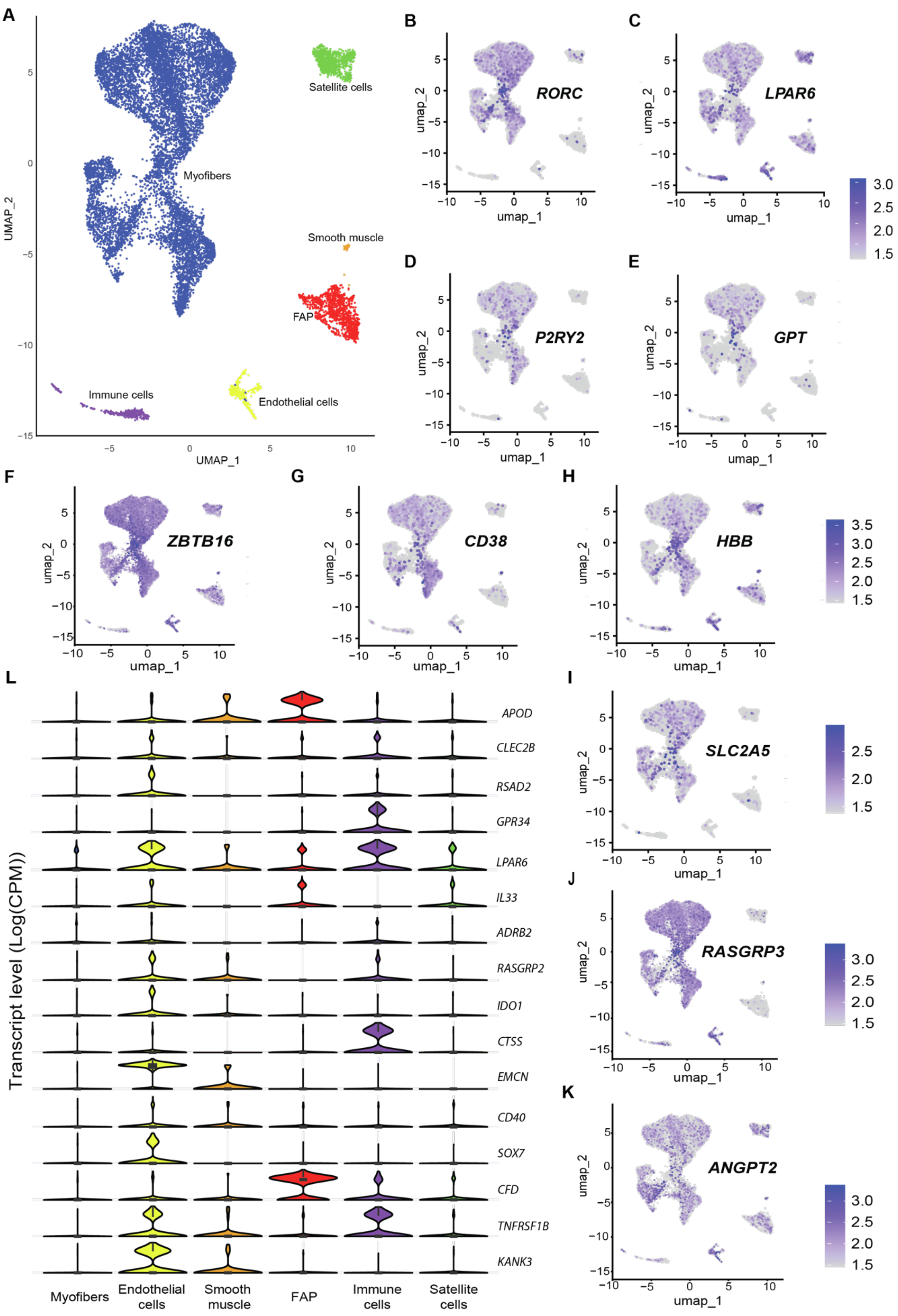
A
Satellite cells
Myofibers
Smooth muscle
FAP
Immune cells
Endothelial cells
UMAP_1
UMAP_2
B
RORC
C
LPAR6
D
P2RY2
E
GPT
F
ZBTB16
G
CD38
H
HBB
I
SLC2A5
J
RASGRP3
K
ANGPT2
umap_1
umap_2
L
APOD
CLEC2B
RSAD2
GPR34
LPAR6
IL33
ADRB2
RASGRP2
IDO1
CTSS
EMCN
CD40
SOX7
CFD
TNFRSF1B
KANK3
Transcript level (Log(CPM))
Myofibers
Endothelial cells
Smooth muscle
FAP
Immune cells
Satellite cells

Figure S2 related to Figure 1: Analysis of single nucleus RNA sequencing data of adult skeletal muscle for the expression of putative myofiber-associated genes (A) UMAP of single nucleus RNA sequencing of adult skeletal muscle sample (Pass, C. G., et al. 2023). Cell identities in clusters are determined by the expression of the marker genes based on the original study and clusters are color-coded for their unique cell types. (B-K) UMAPs highlighting genes from the predicted immune and endothelial system-related gene lists in Fig. 1E, which show high expression in the myofiber cluster. (L) Collection of violin plots showing Z-score transformed transcript levels highlighting the genes suggested to be related to immune and endothelial systems in Figure 1E, but show moderate expression in the myofiber cluster. The presence of a vertical line indicates detected gene expression in a given cluster.

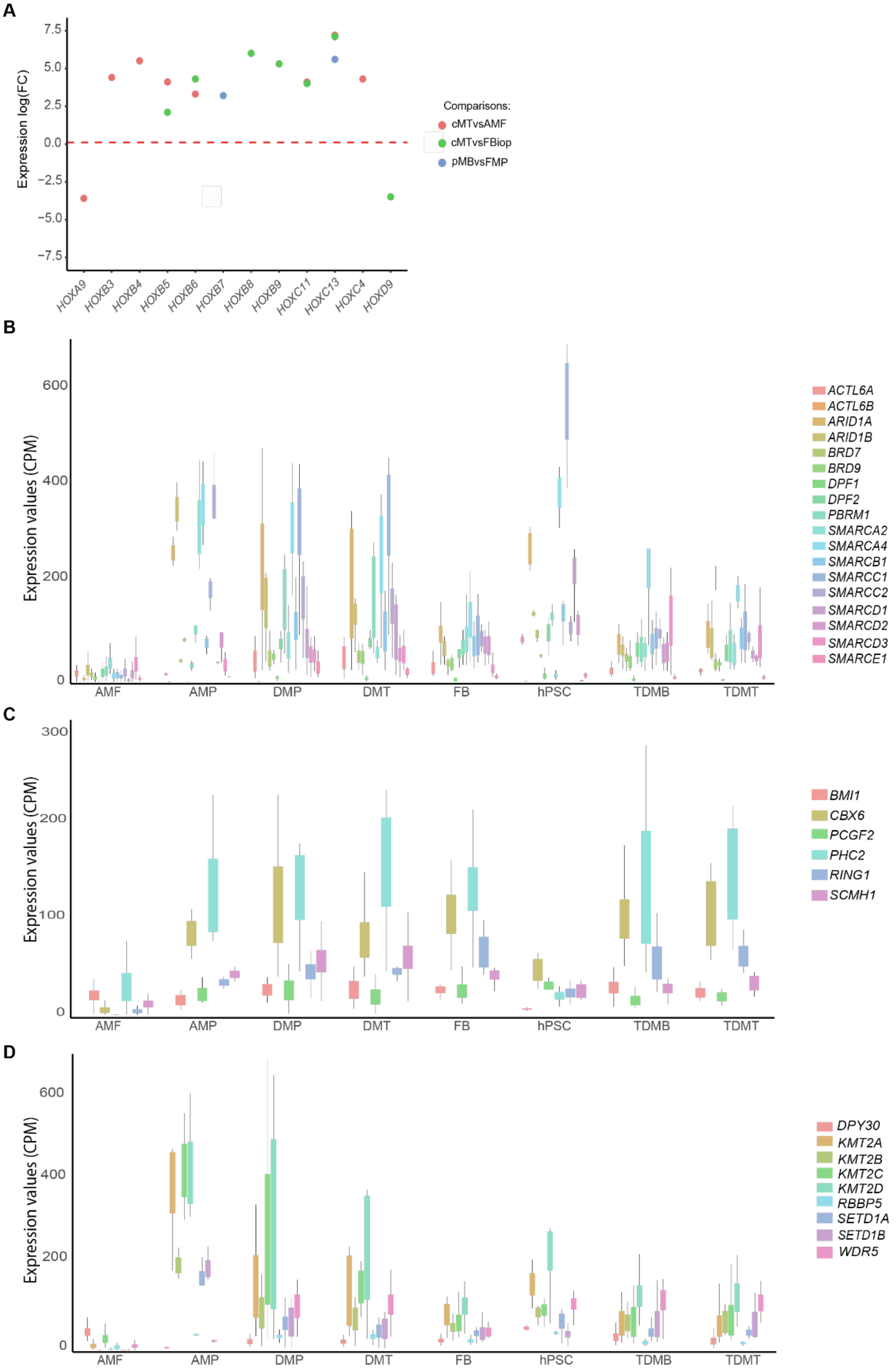

A
Expression log(FC)
7.5
5.0
2.5
0.0
−2.5
−5.0
−7.5
HOXA9
HOXB3
HOXB4
HOXB5
HOXB6
HOXB7
HOXB8
HOXB9
HOXC11
HOXC13
HOXC4
HOXD9
Comparisons:
cMTvsAMF
cMTvsFBiop
pMBvsFMP
B
Expression values (CPM)
600
400
200
0
AMF
AMP
DMP
DMT
FB
hPSC
TDMB
TDMT
ACTL6A
ACTL6B
ARID1A
ARID1B
BRD7
BRD9
DPF1
DPF2
PBRM1
SMARCA2
SMARCA4
SMARCB1
SMARCC1
SMARCC2
SMARCD1
SMARCD2
SMARCD3
SMARCE1
C
Expression values (CPM)
300
200
100
0
AMF
AMP
DMP
DMT
FB
hPSC
TDMB
TDMT
BMI1
CBX6
PCGF2
PHC2
RING1
SCMH1
D
Expression values (CPM)
600
400
200
0
AMF
AMP
DMP
DMT
FB
hPSC
TDMB
TDMT
DPY30
KMT2A
KMT2B
KMT2C
KMT2D
RBBP5
SETD1A
SETD1B
WDR5

40

Figure S3 related to Figure 2: Analysis of transcription and epigenetic factors in immortalized myogenic cell lines, hPSC- or transdifferentiation-derived myogenic cultures and their in vivo references. (A) Dot plot showing the positive and negative standard logarithmic fold change of gene expression for differentially expressed HOX genes in immortalized cell lines in comparison to adult and fetal in vivo references. (B-D) Bar plot showing expression levels of individual active members of SWI/SNF (B), PRC1 (C) and COMPASS/MLL (D) respectively, for all different categories in vivo and in vitro.

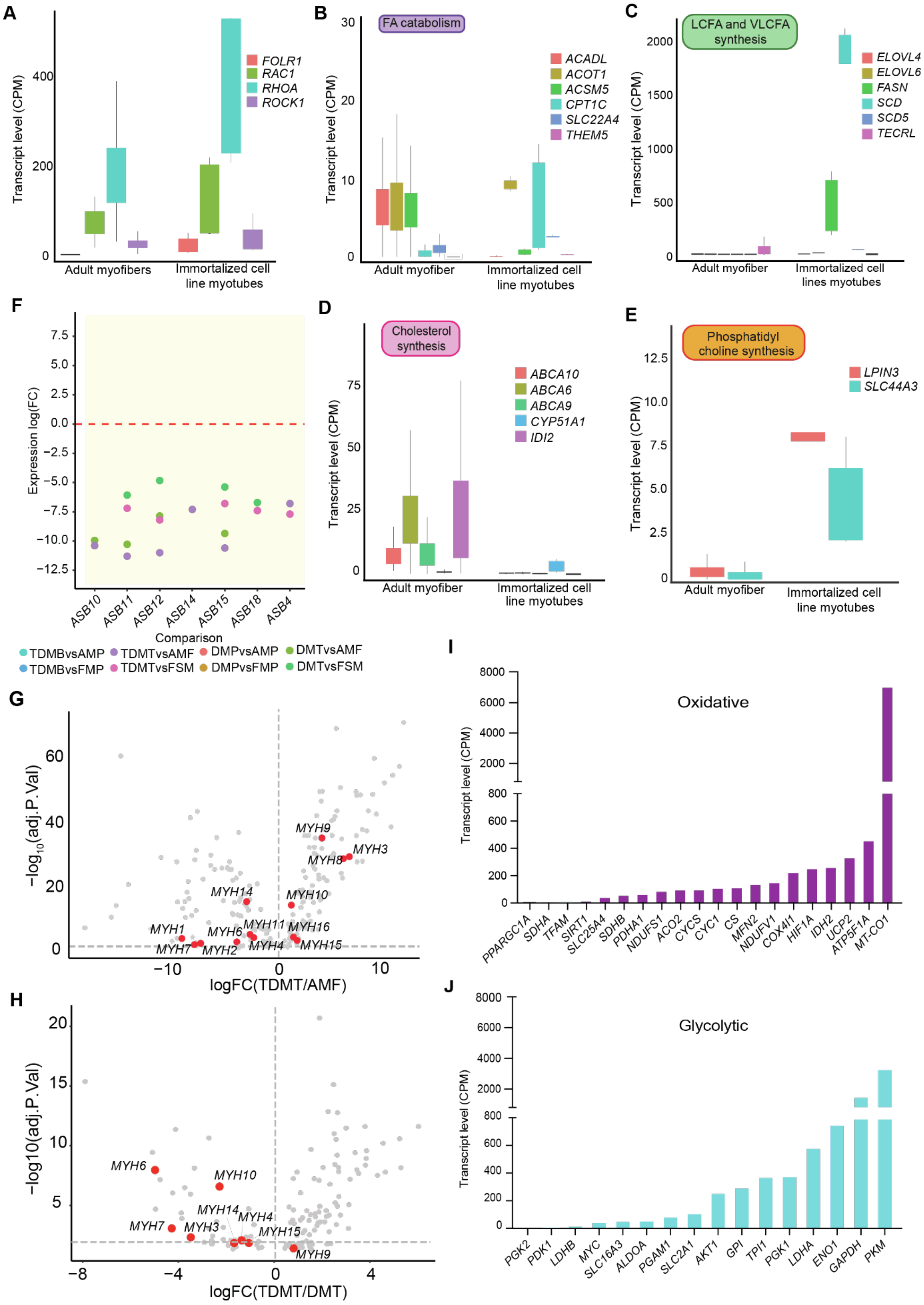
A
Transcript level (CPM)
FOLR1
RAC1
RHOA
ROCK1
Adult myofibers
Immortalized cell line myotubes
B
FA catabolism
Transcript level (CPM)
ACADL
ACOT1
ACSM5
CPT1C
SLC22A4
THEM5
Adult myofiber
Immortalized cell line myotubes
C
LCFA and VLCFA synthesis
Transcript level (CPM)
ELOVL4
ELOVL6
FASN
SCD
SCD5
TECRL
Adult myofiber
Immortalized cell lines myotubes
F
Expression log(FC)
ASB10
ASB11
ASB12
ASB14
ASB15
ASB18
ASB4
Comparison
TDMBvsAMP
TDMTvsAMF
DMPvsAMP
DMTvsAMF
TDMBvsFMP
TDMTvsFSM
DMPvsFMP
DMTvsFSM
D
Cholesterol synthesis
Transcript level (CPM)
ABCA10
ABCA6
ABCA9
CYP51A1
IDI2
Adult myofiber
Immortalized cell line myotubes
E
Phosphatidyl choline synthesis
Transcript level (CPM)
LPIN3
SLC44A3
Adult myofiber
Immortalized cell line myotubes
G
−log10(adj.P.Val)
logFC(TDMT/AMF)
MYH9
MYH3
MYH8
MYH14
MYH10
MYH1
MYH6
MYH11
MYH16
MYH7
MYH2
MYH4
MYH15
H
−log10(adj.P.Val)
logFC(TDMT/DMT)
MYH6
MYH10
MYH14
MYH4
MYH7
MYH3
MYH15
MYH9
I
Oxidative
Transcript level (CPM)
PPARGC1A
SDHA
TFAM
SIRT1
SLC25A4
SDHB
PDHA1
NDUFS1
ACO2
CYCS
CYC1
CS
MFN2
NDUFV1
COX4I1
HIF1A
IDH2
UCP2
ATP5F1A
MT-CO1
J
Glycolytic
Transcript level (CPM)
PGK2
PDK1
LDHB
MYC
SLC16A3
ALDOA
PGAM1
SLC2A1
AKT1
GPI
TPI1
PGK1
LDHA
ENO1
GAPDH
PKM

Figure S4 related to Figure 3: Metabolic and fiber type signatures in immortalized myogenic cell lines, hPSC-derived differentiated and fibroblast-derived transdifferentiated myogenic cultures. (A) Bar plot showing average transcript levels (CPM) of folic acid cycle members for AMF and iMT. (B-E) Bar plots displaying average transcript levels (CPM) of the members of fatty acid and lipid metabolism subprocesses for AMF and iMT: fatty acid catabolism (B), long chain fatty acid and very-long-chain fatty acid synthesis (C), cholesterol synthesis (D) and phosphatidyl choline synthesis (E). (F) Dot plot showing the standard logarithmic fold change of expression of ASB family of E3 ubiquitin ligases across the indicated comparisons. (G-H) Volcano plot showing the myogenic DEGs between TDMT and the AMF (G), and TDMT and DMT (H), highlighting different Myosin Heavy Chains (I-J) Bar plot showing average expression levels (CPM) of genes implicated in glycolytic or oxidative energy metabolism for TDMT.

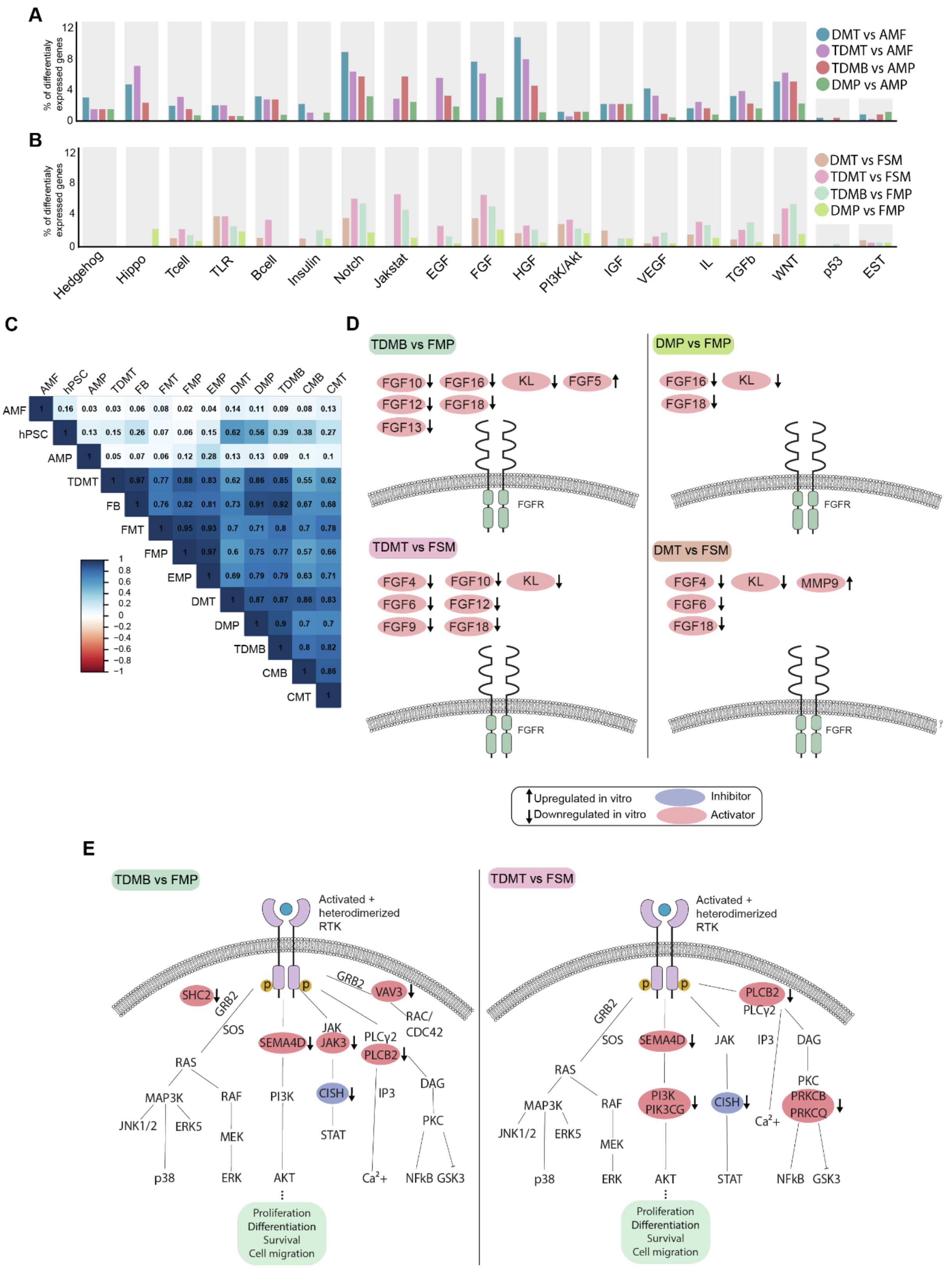
A
% of differentialy expressed genes
12
8
4
0
DMT vs AMF
TDMT vs AMF
TDMB vs AMP
DMP vs AMP
B
% of differentialy expressed genes
12
8
4
0
DMT vs FSM
TDMT vs FSM
TDMB vs FMP
DMP vs FMP
Hedgehog
Hippo
Tcell
TLR
Bcell
Insulin
Notch
Jakstat
EGF
FGF
HGF
PI3K/Akt
IGF
VEGF
IL
TGFb
WNT
p53
EST
C
AMF hPSC AMP TDMT FB FMT FMP EMP DMT DMP TDMB CMB CMT
AMF 1 0.16 0.03 0.03 0.06 0.08 0.02 0.04 0.14 0.11 0.09 0.08 0.13
hPSC 1 0.13 0.15 0.26 0.07 0.06 0.15 0.62 0.56 0.39 0.38 0.27
AMP 1 0.05 0.07 0.06 0.12 0.28 0.13 0.13 0.09 0.1 0.1
TDMT 1 0.97 0.77 0.88 0.83 0.62 0.86 0.85 0.55 0.62
FB 1 0.76 0.82 0.81 0.73 0.91 0.92 0.67 0.68
FMT 1 0.95 0.93 0.7 0.71 0.8 0.7 0.78
FMP 1 0.97 0.6 0.75 0.77 0.57 0.66
EMP 1 0.69 0.79 0.79 0.63 0.71
DMT 1 0.87 0.87 0.86 0.83
DMP 1 0.9 0.7 0.7
TDMB 1 0.8 0.82
CMB 1 0.86
CMT 1
1
0.8
0.6
0.4
0.2
0
−0.2
−0.4
−0.6
−0.8
−1
D
TDMB vs FMP
FGF10 ↓ FGF16 ↓ KL ↓ FGF5 ↑
FGF12 ↓ FGF18 ↓
FGF13 ↓
FGFR
DMP vs FMP
FGF16 ↓ KL ↓
FGF18 ↓
FGFR
TDMT vs FSM
FGF4 ↓ FGF10 ↓ KL ↓
FGF6 ↓ FGF12 ↓
FGF9 ↓ FGF18 ↓
FGFR
DMT vs FSM
FGF4 ↓ KL ↓ MMP9 ↑
FGF6 ↓
FGF18 ↓
FGFR
↑ Upregulated in vitro
↓ Downregulated in vitro
Inhibitor
Activator
E
TDMB vs FMP
Activated + heterodimerized RTK
p p
SHC2 ↓
GRB2
SOS
GRB2
VAV3 ↓
RAC/ CDC42
SEMA4D ↓
JAK
JAK3 ↓
PLCγ2
PLCB2 ↓
RAS
MAP3K
RAF
PI3K
CISH ↓
IP3
DAG
JNK1/2
ERK5
MEK
STAT
PKC
p38
ERK
AKT
Ca2+
NFkB GSK3
Proliferation
Differentiation
Survival
Cell migration
TDMT vs FSM
Activated + heterodimerized RTK
p p
PLCB2 ↓
PLCγ2
GRB2
SOS
SEMA4D ↓
JAK
IP3
DAG
RAS
PKC
MAP3K
RAF
PI3K
PIK3CG ↓
CISH ↓
PRKCB
PRKCQ ↓
Ca2+
JNK1/2
ERK5
MEK
p38
ERK
AKT
STAT
NFkB GSK3
Proliferation
Differentiation
Survival
Cell migration

83

Figure S5 related to Figure 4: Analysis of aberrant expression of signaling pathway members in the in vitro models. (A-B) Bar plots showing the number of significantly up- and downregulated members of each signaling pathway, which passed strict median CPM filtering for in vitro comparisons to adult (A) and fetal (B) references. (C) Pearson correlation matrix for median CPM of signaling pathway genes used in Figure 4C. Blue signifies positive correlation, whereas red indicates a negative correlation. (D) Individual schematics showcasing the differentially expressed ligands of the FGF pathway for transdifferentiated myoblasts compared to fetal myogenic progenitors (upper left), DMP compared to FMP (upper right), TDMT compared to FSM (lower left) and DMT compared to FSM (lower right). (E) Schematics highlighting the differentially expressed members of the downstream RTK signaling cascades for TDMB compared to FMP (left) and TDMT compared FSM (right).

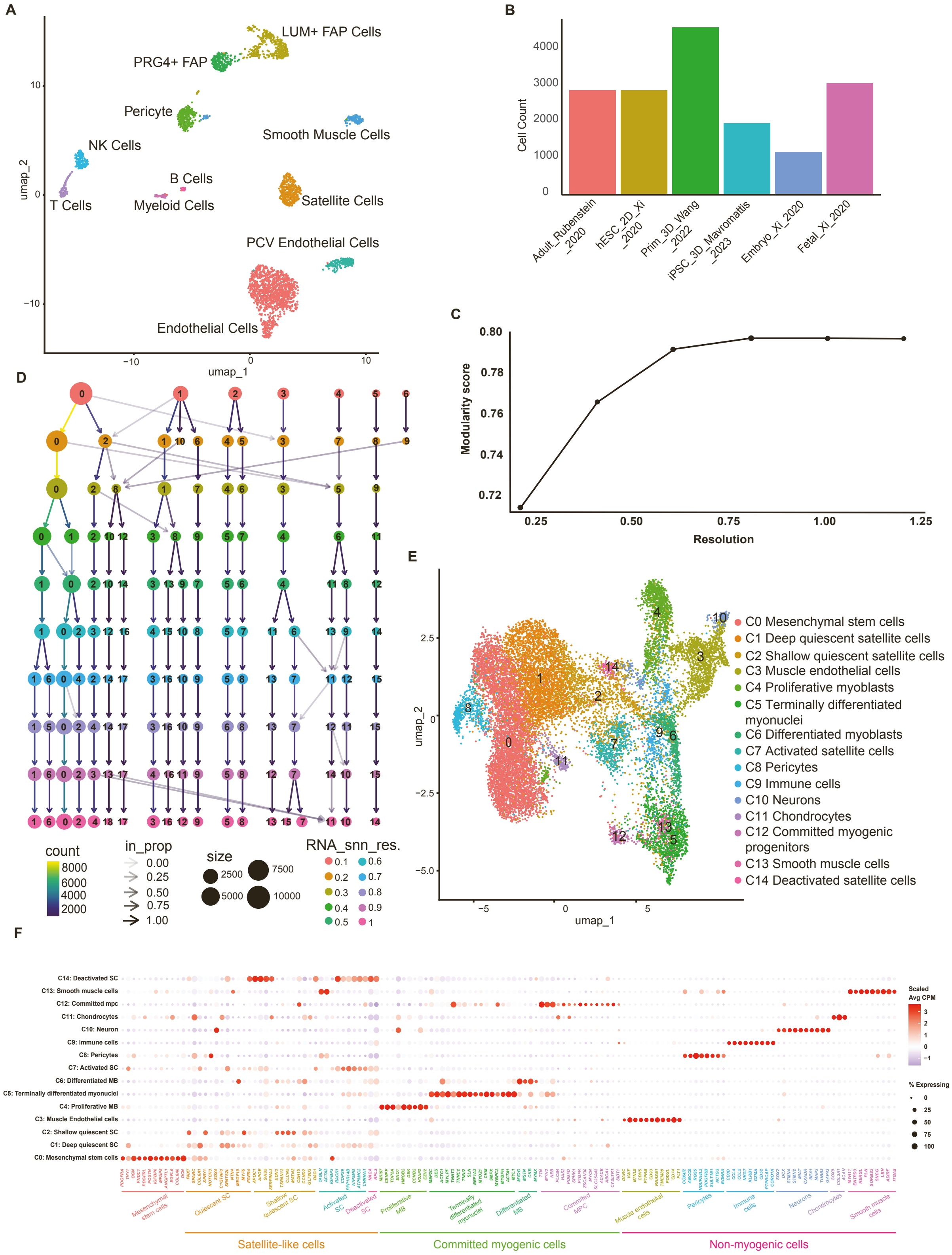
A
LUM+ FAP Cells
PRG4+ FAP
Pericyte
Smooth Muscle Cells
NK Cells
B Cells
T Cells
Myeloid Cells
Satellite Cells
PCV Endothelial Cells
Endothelial Cells
umap_1
umap_2
B
Cell Count
Adult_Rubenstein_2020
hESC_2D_Xi_2020
Prim_3D_Wang_2022
iPSC_3D_Mavromattis_2023
Embryo_Xi_2020
Fetal_Xi_2020
C
Modularity score
Resolution
D
count
in_prop
size
RNA_snn_res.
E
C0 Mesenchymal stem cells
C1 Deep quiescent satellite cells
C2 Shallow quiescent satellite cells
C3 Muscle endothelial cells
C4 Proliferative myoblasts
C5 Terminally differentiated myonuclei
C6 Differentiated myoblasts
C7 Activated satellite cells
C8 Pericytes
C9 Immune cells
C10 Neurons
C11 Chondrocytes
C12 Committed myogenic progenitors
C13 Smooth muscle cells
C14 Deactivated satellite cells
F
C14: Deactivated SC
C13: Smooth muscle cells
C12: Committed mpc
C11: Chondrocytes
C10: Neuron
C9: Immune cells
C8: Pericytes
C7: Activated SC
C6: Differentiated MB
C5: Terminally differentiated myonuclei
C4: Proliferative MB
C3: Muscle Endothelial cells
C2: Shallow quiescent SC
C1: Deep quiescent SC
C0: Mesenchymal stem cells
Scaled Avg CPM
% Expressing
Satellite-like cells
Committed myogenic cells
Non-myogenic cells

Figure S6 related to Figure 5: Integration of in vitro and in vivo scRNAseq datasets and characterization of the *PAX7*$^{+}$ clusters. (A) UMAP showing the clustering of a scRNAseq adult skeletal muscle biopsy and highlighting the lack of myofiber-associated nuclei when compared to Figure S2A that shows snRNAseq analysis of another skeletal muscle biopsy. (B) Bar plot, showing the number of cells per scRNAseq per dataset. (C) Modularity score plotted against resolution to identify the optimal resolution. (D) Force-directed cluster tree to identify optimal resolution, for which cluster identity is stable without overfragmentation. (E) Annotated UMAP of the integrated dataset of 15 clusters. (F) Dot plot showing expression of marker gene sets per cell type for 15 clusters.

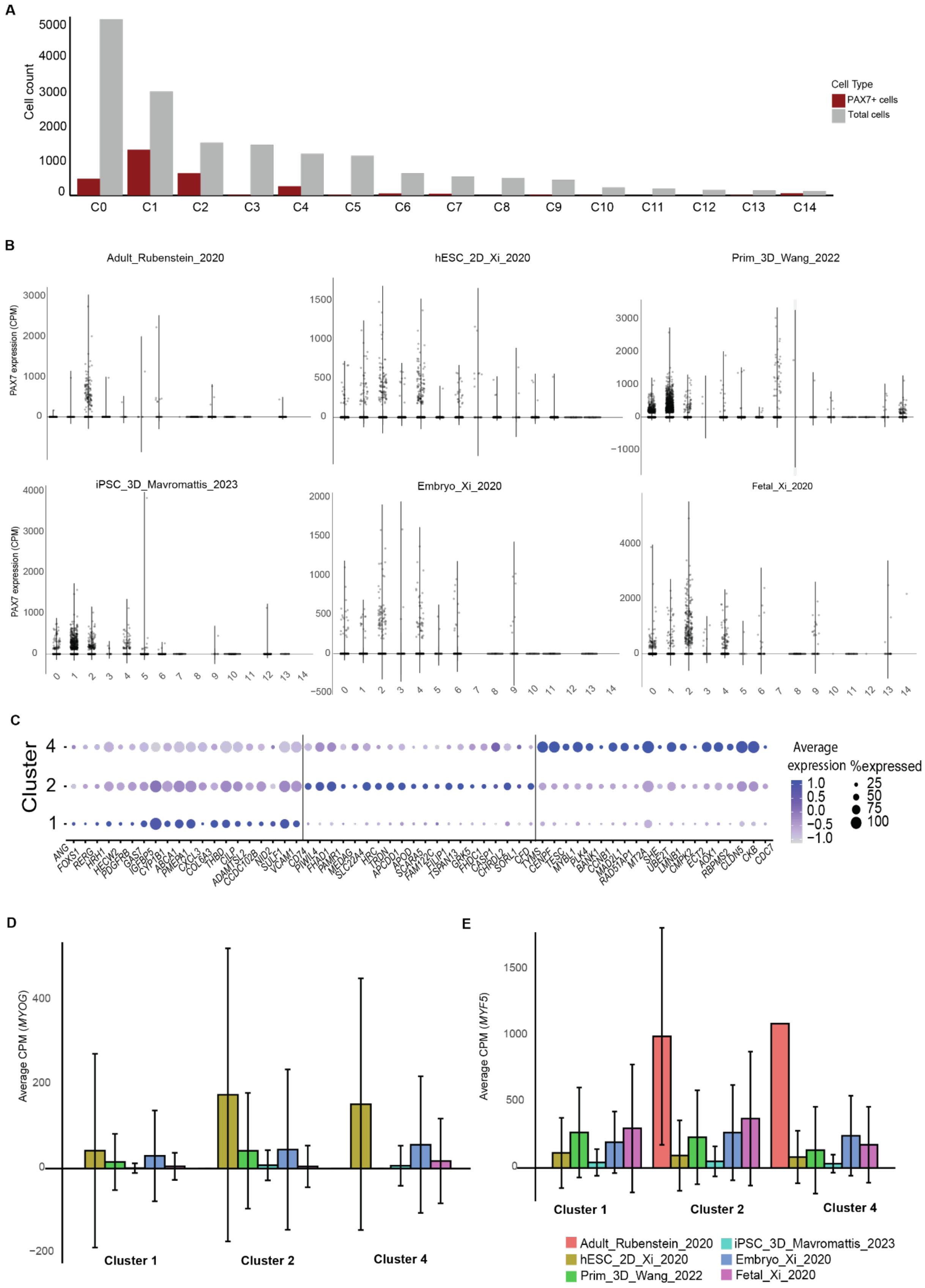

A
Cell count
5000
4000
3000
2000
1000
0
C0 C1 C2 C3 C4 C5 C6 C7 C8 C9 C10 C11 C12 C13 C14
Cell Type
PAX7+ cells
Total cells
B
Adult_Rubenstein_2020
hESC_2D_Xi_2020
Prim_3D_Wang_2022
iPSC_3D_Mavromattis_2023
Embryo_Xi_2020
Fetal_Xi_2020
PAX7 expression (CPM)
C
Cluster
Average expression
%expressed
ANG FOXS1 RERG HRH1 HECW2 PDGFRB GAS7 IGFBP5 CYP1B1 ABCA1 PMEPA1 CXCL3 COL6A3 THBD CILP ADAMTSL2 CDC102B NID2 SULF1 VCAM1 CD74 PIWIL4 FHAD1 PAMR1 MEDAG SLC22A4 HRC TRDN APCDD1 APOD SCARA5 FAM122C FILIP1 TSPAN13 GRK5 FHDC1 CASP1 CHRDL2 SORL1 CFD TYMS CENPF TESC MYBL1 PLK4 BANK1 CCNB1 MAD2L1 RAD51AP1 MT2A SHE UBE2T LMNB1 CMPK2 ECT2 AOX1 RBPMS2 CLDN5 CKB CDC7
D
Average CPM (MYOG)
Cluster 1
Cluster 2
Cluster 4
E
Average CPM (MYF5)
Cluster 1
Cluster 2
Cluster 4
Adult_Rubenstein_2020
hESC_2D_Xi_2020
Prim_3D_Wang_2022
iPSC_3D_Mavromattis_2023
Embryo_Xi_2020
Fetal_Xi_2020

Figure S7 related to Figure 5: Analysis of individual clusters of the integrated scRNAseq dataset. (A) Number of *PAX7*$^{+}$ and total number of cells per cluster of the integrated dataset. (B) Violin plot showing distribution of *PAX7* expression (CPM) per study per cluster. (C) Dot plot showing the average CPM expression of the top 20 highly variable genes for cluster 1, 2 and 4. (D-E) Bar plot showing the average CPM expression of *MYOG* per study for cluster 1, 2 and 4 (D) and *MYF5* per study per cluster (E).
